# KLF11 protects against abdominal aortic aneurysm through inhibition of endothelial cell dysfunction

**DOI:** 10.1172/jci.insight.141673

**Published:** 2021-03-08

**Authors:** Guizhen Zhao, Ziyi Chang, Yang Zhao, Yanhong Guo, Haocheng Lu, Wenying Liang, Oren Rom, Huilun Wang, Jinjian Sun, Tianqing Zhu, Yanbo Fan, Lin Chang, Bo Yang, Minerva T. Garcia-Barrio, Y. Eugene Chen, Jifeng Zhang

**Affiliations:** 1 Department of Internal Medicine, University of Michigan Medical Center, Ann Arbor, Michigan, USA.; 2 Department of Pathology, China-Japan Friendship Hospital, Beijing, China.; 3 Department of Cardiovascular Medicine, The Second Xiangya Hospital, Central South University, Changsha, China.; 4 Department of Cancer Biology, College of Medicine, University of Cincinnati, Cincinnati, Ohio. USA.; 5 Department of Cardiac Surgery, University of Michigan Medical Center, Ann Arbor, Michigan, USA.

**Keywords:** Vascular Biology, Cardiovascular disease

## Abstract

Abdominal aortic aneurysm (AAA) is a life-threatening degenerative vascular disease. Endothelial cell (EC) dysfunction is implicated in AAA. Our group recently demonstrated that Krüppel-like factor 11 (KLF11) plays an essential role in maintaining vascular homeostasis, at least partially through inhibition of EC inflammatory activation. However, the functions of endothelial KLF11 in AAA remain unknown. Here we found that endothelial KLF11 expression was reduced in the ECs from human aneurysms and was time dependently decreased in the aneurysmal endothelium from both elastase- and *Pcsk9*/AngII-induced AAA mouse models. KLF11 deficiency in ECs markedly aggravated AAA formation, whereas EC-selective KLF11 overexpression markedly inhibited AAA formation. Mechanistically, KLF11 not only inhibited the EC inflammatory response but also diminished MMP9 expression and activity and reduced NADPH oxidase 2–mediated production of reactive oxygen species in ECs. In addition, KLF11-deficient ECs induced smooth muscle cell dedifferentiation and apoptosis. Overall, we established endothelial KLF11 as a potentially novel factor protecting against AAA and a potential target for intervention in aortic aneurysms.

## Graphical abstract

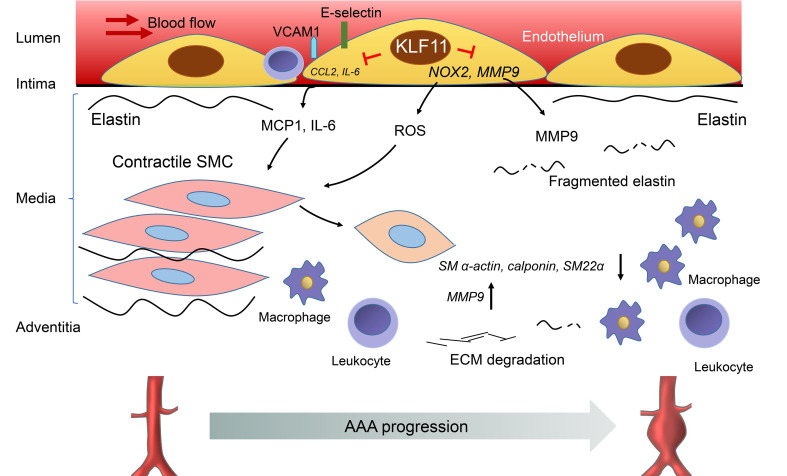

## Introduction

Abdominal aortic aneurysm (AAA) is an irreversible degenerative disease, and its rupture has a mortality rate up to 90% (1, 2). The risk factors for AAA include age, male sex, smoking, hypertension, and atherosclerosis (1, 2). AAA develops because of a combination of vascular inflammation, excessive oxidative stress, and maladaptive aortic wall remodeling (2, 3). At present, only 10% of the patients are eligible for open surgical repair or invasive endovascular aneurysm repair, while drug-based therapies are still lacking (1, 3). There is an urgent need for a better understanding of the mechanisms underlying AAA development to help design novel, noninvasive therapeutic approaches.

Healthy endothelium is critical in maintaining vascular homeostasis (4, 5) through regulation of vascular tone, cellular adhesion, and vascular smooth muscle cell (VSMC) homeostasis. However, prolonged exposure to cardiovascular risk factors, like smoking, hyperlipidemia, and proinflammatory factors, induces endothelial cell (EC) dysfunction and subsequently exacerbates vascular diseases, such as vascular inflammation (6) and atherosclerosis (5). ECs can also increase oxidative stress in the vessel wall by impaired nitric oxide bioavailability due to endothelial dysfunction and NADPH oxidase (NOX) upregulation (7). Moreover, endothelial dysfunction has been found to trigger vascular remodeling by releasing proteases or recruiting immune cells into the medial layer (8, 9). Current evidence (10, 11) supports the notion that pathological changes in ECs are likely essential steps to initiate the process of AAA formation. Therefore, investigation of EC dysfunction and the interaction between the endothelium and medial smooth muscle cells (SMCs) during AAA development may provide a deeper understanding of AAA pathology toward novel targeted interventions.

Krüppel-like factors, a family of zinc-finger–containing transcription factors, have been implicated in many biological processes, including cell proliferation, differentiation, and apoptosis (12, 13). Krüppel-like factor 11 (KLF11) is a member of the KLF family with high expression in various human tissues, including the vasculature (12). KLF11 is a vasoprotective factor and plays an essential role in maintaining vascular homeostasis (12, 14, 15). Moreover, our prior studies demonstrated that KLF11 cooperates with peroxisome proliferator–activated receptor γ to reduce ischemic cerebral vascular endothelium damage (16) and inhibits EC inflammatory activation in the presence of proinflammatory stimuli (14). Noteworthy, despite emerging data from clinical and animal studies suggesting that EC dysfunction is also highly associated with AAA, the potential effect and underlying mechanism of endothelial KLF11 in AAA development remains to be addressed. Consequently, in the present study, we sought to define whether KLF11 has a protective role in AAA formation by improving endothelial cell functions and contributing to VSMC homeostasis.

## Results

### KLF11 is reduced in aneurysmal endothelium.

**Figure 1:**
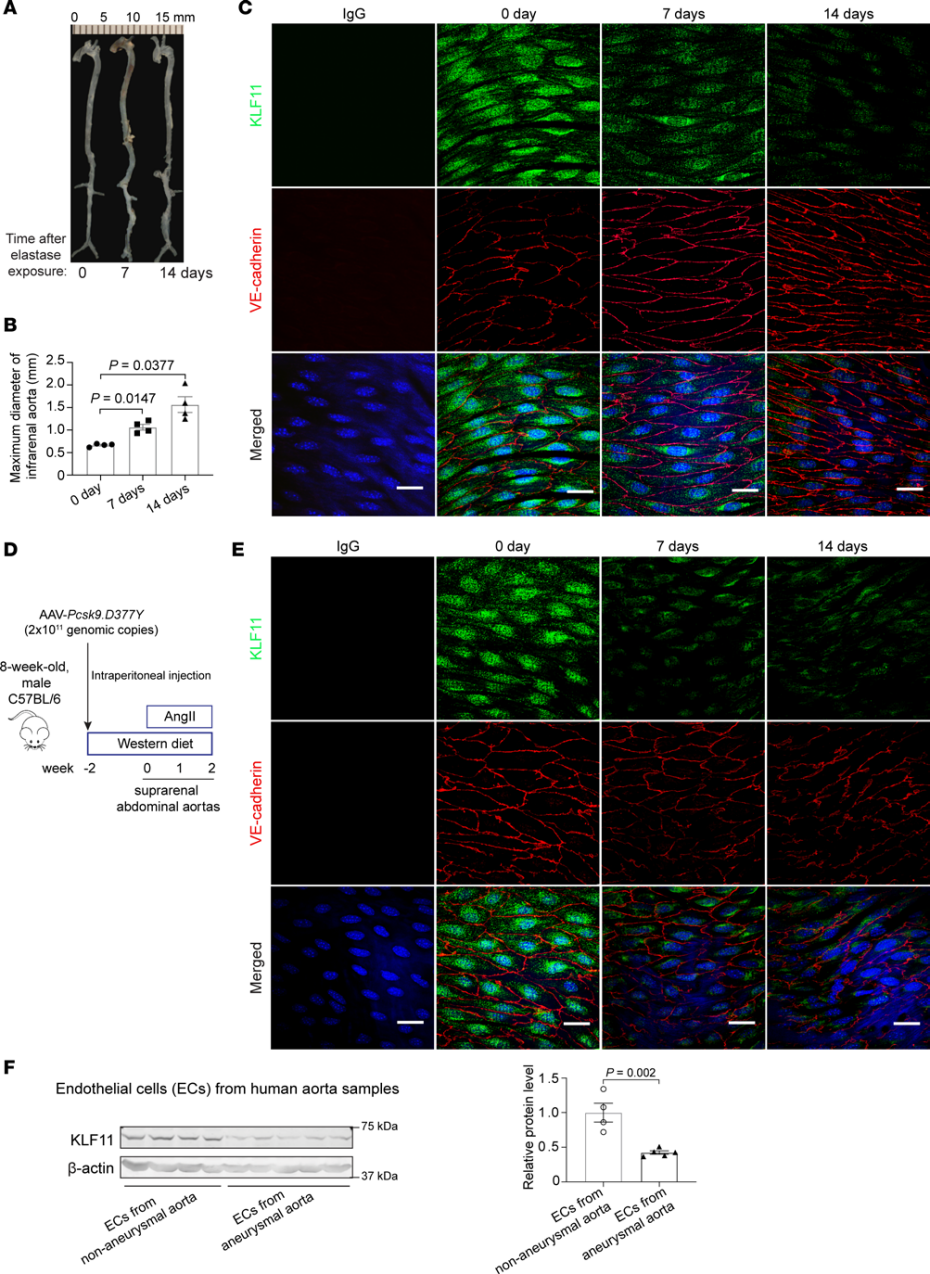
KLF11 is reduced in the ECs of mouse and human aortic aneurysms. (**A**–**C**) Elastase-induced AAA model in 10-week-old C57BL/6 male mice (*n* = 4/group). Representative morphology of aortas (**A**) and quantification of maximal infrarenal aortic diameters (**B**). En face immunofluorescence staining of KLF11 (shown in green) and VE-cadherin (shown in red, EC marker) in the endothelium of elastase-treated infrarenal aortas (**C**) and AngII-infused suprarenal abdominal aortas (**D** and **E**) from the indicated end time points. Nuclei stained by DAPI are shown in blue. Scale bar: 20 μm. (**F**) Western blot analysis of KLF11 expression in the ECs from human normal (*n* = 4) and aneurysmal aortas (*n* = 5). Data are presented as mean ± SEM. One-way ANOVA followed by Tukey’s post hoc for **B**, Student’s 2-tailed *t* test for **E**.

To explore the possible relationship between KLF11 and AAA, we adopted 2 distinct murine AAA models (17–19): the elastase-induced AAA model and the proprotein convertase subtilisin/kexin type 9 and angiotensin II–induced (*Pcsk9*/AngII-induced) AAA model. In the elastase model (Figure 1, A and B), the progressive increase in the internal diameter of the infrarenal aorta was directly associated with a time-dependent reduction in endothelial KLF11 expression as assessed by en face immunofluorescence staining (Figure 1C). The progressive downregulation of KLF11 was mirrored in the endothelium of the suprarenal abdominal aortas after AngII infusion (Figure 1, D and E). Those findings were consistent with the observation that the protein abundance of KLF11 was significantly reduced in the endothelial cells of aortic samples from human aortic aneurysms (Figure 1F and Supplemental Table 1; supplemental material available online with this article; https://doi.org/10.1172/jci.insight.141673DS1). Thus, these results indicate that KLF11 downregulation is involved in the pathology of AAA.

### Endothelial KLF11 deficiency aggravates AAA formation.

**Figure 2:**
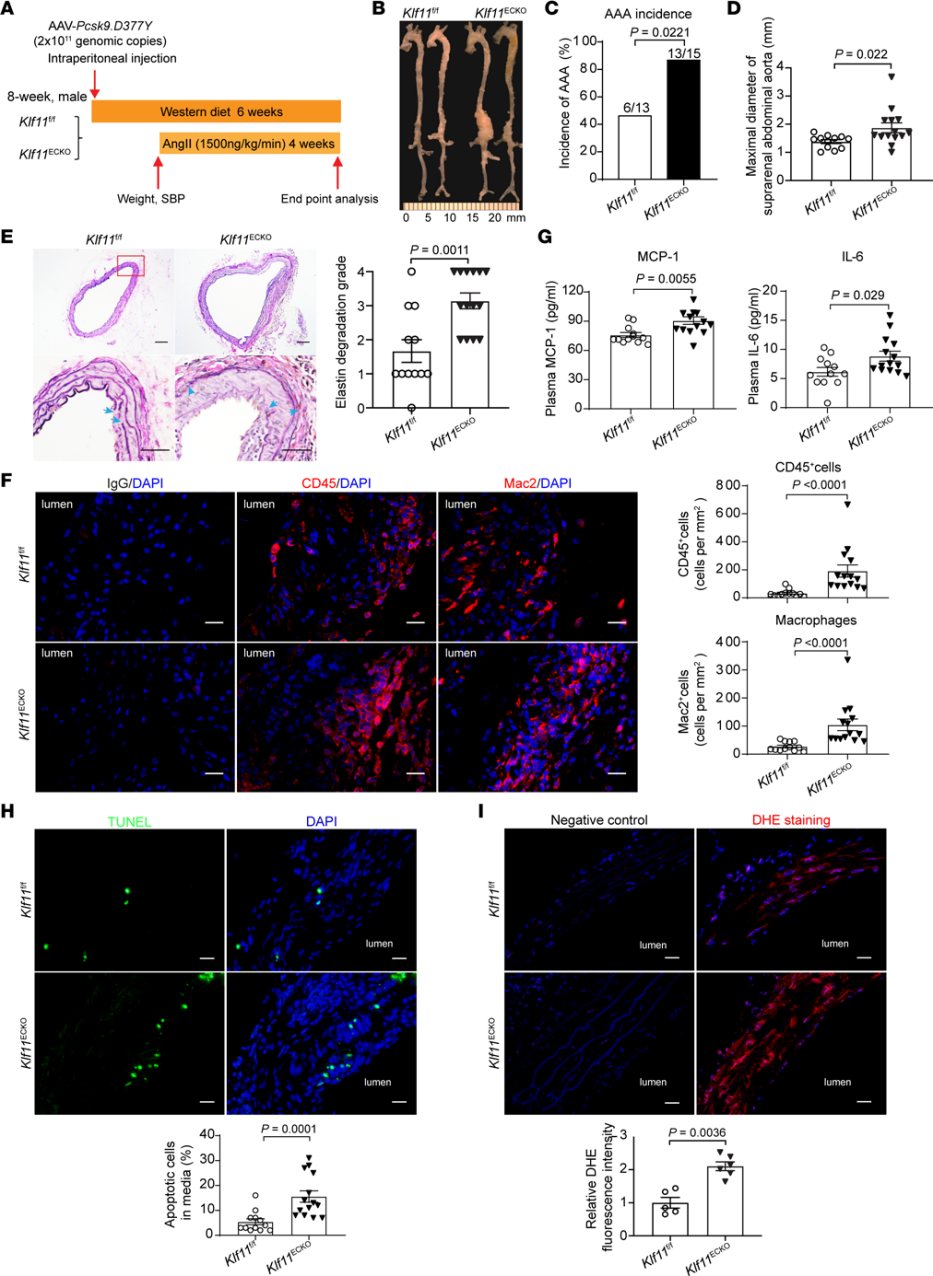
Endothelial cell–specific KLF11 depletion aggravates *Pcsk9*/AngII-induced AAA. The *Pcsk9*/AngII-induced AAA model was performed on 8-week-old males with *Klf11*^ECKO^ (*n* = 15) and their littermate *Klf11*^fl/fl^ (*n* = 13) mice. (**A**) Schematics of *Pcsk9*/AngII-induced AAA model. (**B**) Representative morphology of aortas from AngII-infused *Klf11*^fl/fl^ and *Klf11*^ECKO^ mice. (**C**) Incidence of AAA. (**D**) Maximal diameters of suprarenal abdominal aortas (SAAs) from AngII-infused *Klf11*^fl/fl^ (*n* = 12) and *Klf11*^ECKO^ (*n* = 14) mice. (**E**) Representative Verhoeff-Van Gieson (VVG) staining and quantification of elastin degradation in SAAs from AngII-infused *Klf11*^fl/fl^ (*n* = 12) and *Klf11*^ECKO^ (*n* = 14) mice. Scale bar: 50 μm. (**F**) Representative immunofluorescence staining and quantification of leukocyte (CD45^+^) and macrophage (Mac2^+^) infiltration in the aortic wall of SAAs from AngII-infused *Klf11*^fl/fl^ (*n* = 12) and *Klf11*^ECKO^ (*n* = 14) mice. Scale bar: 20 μm. (**G**) ELISA analysis of MCP-1 and IL-6 in the plasma from AngII-infused *Klf11*^fl/fl^ (*n* = 12) and *Klf11*^ECKO^ (*n* = 14) mice. (**H**) Representative TUNEL staining (green) and quantification of apoptotic cells in the media of SAAs from AngII-infused *Klf11*^fl/fl^ (*n* = 12) and *Klf11*^ECKO^ (*n* = 14) mice. Scale bar: 20 μm. (**I**) Representative DHE staining (red) and quantification of ROS production in the aortic wall of SAAs from AngII-infused *Klf11*^fl/fl^ (*n* = 5) and *Klf11*^ECKO^ (*n* = 6). Scale bar: 20 μm. Data are presented as mean ± SEM. χ^2^ test (**C**), Student’s 2-tailed *t* test (**D**, **G**, and **I**), Mann-Whitney *U* test (**E**, **F**, and **H**).

To study the role of KLF11 in ECs during AAA development in vivo, we generated EC-specific *Klf11*-knockout (*Klf11*^ECKO^) mice by crossbreeding *Klf11*-floxed mice (*Klf11*^fl/fl^) with B6.Cg-Tg(*Tek*-Cre)1Ywa/J (*Tie2*-Cre) mice (Supplemental Figure 1A). The *Klf11* knockout in ECs was determined by Western blot and quantitative PCR (qPCR), and no significant differences in the expression of other *Klf*s were noted (Supplemental Figure 1, B and C). Next, 8-week-old male *Klf11*^ECKO^ (*n* = 15) and littermate *Klf11*^fl/fl^ (*n* = 13) mice were subjected to *Pcsk9*/AngII-induced AAA model (Figure 2A). We observed 1 out of 13 (7.7%) in the *Klf11*^fl/fl^ group and 1 out of 15 (6.7%) in the *Klf11*^ECKO^ group died due to rupture of AAA. The survival rate, body weight, blood pressure, and plasma lipid profiles were comparable between the 2 groups (Supplemental Figure 1, D–G). Meanwhile, AngII infusion did not affect the endothelium integrity assessed by immunofluorescence staining of 2 EC markers, CD31 and VE-cadherin, in the AAA region (Supplemental Figure 1H). Nevertheless, endothelial *Klf11* deficiency markedly increased the incidence of AAA (86.67%) and the maximal diameters of the suprarenal abdominal aorta (1.872 ± 0.175 mm) compared with those in *Klf11*^fl/fl^ mice (AAA incidence of 46.15% and maximal diameter of 1.383 ± 0 .067 mm) after AngII infusion (Figure 2, B–D). Endothelial *Klf11* knockout markedly enhanced elastin degradation and matrix metallopeptidase 9 (MMP9) expression in the aortic sections, particularly in the endothelium (Figure 2E and Supplemental Figure 1I). Moreover, leukocyte (CD45^+^) and macrophage (Mac2^+^) infiltration to the aortic wall and the concentration of plasma monocyte chemoattractant protein-1 (MCP-1) and IL-6 were markedly increased in *Klf11*^ECKO^ mice compared with *Klf11*^fl/fl^ mice (Figure 2, F and G). Additionally, endothelial *Klf11* deficiency significantly increased VSMC apoptosis assessed by TUNEL staining and superoxide production assessed by dihydroethidium (DHE) staining in the aortic wall of *Pcsk9*/AngII-induced AAA (Figure 2, H and I).

We also performed elastase-induced infrarenal AAA on 8- to 12-week-old male *Klf11*^fl/fl^ and *Klf11*^ECKO^ mice. Fourteen days after elastase exposure, the body weight, blood pressure, plasma lipid profiles, and MCP-1 were comparable between the 2 groups (Supplemental Figure 2, A–D). Consistent with our findings in the *Pcsk9*/AngII-induced AAA model, *Klf11*^ECKO^ mice exhibited enhanced abdominal aortic enlargement, elastin degradation, as well as leukocyte and macrophage accumulation in the aortic walls, compared with *Klf11*^fl/fl^ mice (Supplemental Figure 2, E–H). These data support that loss of KLF11 promotes AAA.

### KLF11 overexpression in ECs attenuates AAA formation.

**Figure 3:**
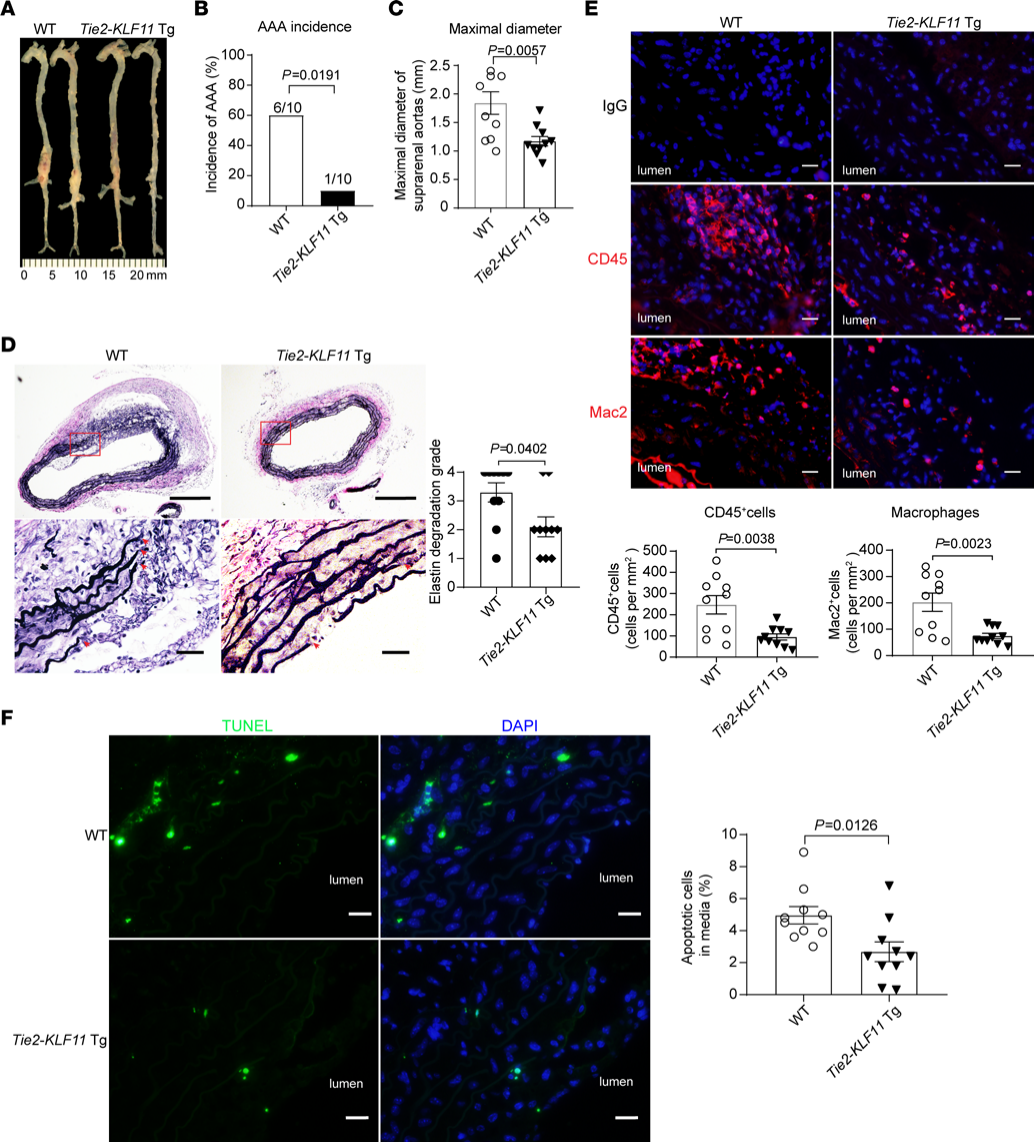
EC-selective overexpression of KLF11 attenuates *Pcsk9*/AngII-induced AAA. The *Pcsk9*/AngII-induced AAA model was performed on 10-week-old male EC-selective *KLF11* transgenic mice (*Tie2-KLF11*–Tg, *n* = 12) and littermate control mice (WT, *n* = 13). (**A**) Representative morphology of aortas from AngII-infused WT and *Tie2-KLF11*–Tg mice. (**B**) Incidence of AAA. (**C**) Maximal diameters of SAAs. (**D**) Representative VVG staining and quantification of elastin degradation in SAAs from WT and *Tie2-KLF11*–Tg mice (*n* = 10/group). Scale bar: 200 μm for whole aortic sections, 20 μm for magnified areas. (**E**) Representative immunofluorescence staining and quantification of leukocyte (CD45^+^) and macrophage (Mac2^+^) infiltration in the aortic wall of SAAs from WT and *Tie2-KLF11*–Tg mice (*n* = 10/group). Scale bar: 20 μm. (**F**) Representative TUNEL staining (green) and quantification of apoptotic cells in the media of SAAs from WT and *Tie2-KLF11*–Tg mice (*n* = 10/group). Scale bar: 20 μm. Data are presented as mean ± SEM. χ^2^ test (**B**), Student’s 2-tailed *t* test (**C**, **E**, and **F**), Mann-Whitney *U* test (**D**).

To further evaluate the protective role of endothelial KLF11 in AAA, we generated EC-selective *KLF11*-transgenic mice (*Tie2-KLF11*–Tg mice) and validated KLF11 overexpression in ECs through Western blot (Supplemental Figure 3, A and B). Next, we performed the *Pcsk9*/AngII-induced AAA model on male *Tie2-KLF11*–Tg mice and their littermate WT mice. No significant differences were found regarding survival rate (3 mice in WT group and 2 mice in *Tie2-KLF11*–Tg group died due to rupture of the thoracic aorta), systolic blood pressure, body weight, and plasma lipid profiles between the 2 groups (Supplemental Figure 3, C–F). However, KLF11 overexpression in ECs significantly reduced AAA incidence (WT, 60% vs. *Tie2-KLF11*–Tg, 10%, Figure 3, A and B). Concomitantly, the maximal suprarenal aortic diameter (WT, 1.841 ± 0.137 mm vs. *Tie2-KLF11*–Tg, 1.172 ± 0.063 mm), elastin degradation, MMP9 expression in the endothelium, and leukocyte and macrophage accumulation in the aortic wall were significantly attenuated in *Tie2-KLF11*–Tg mice compared with WT mice (Figure 3, C–E, and Supplemental Figure 3G). Additionally, KLF11 overexpression in ECs significantly reduced VSMC apoptosis in the aortic wall (Figure 3F).

Next, the elastase-induced AAA model was also performed on the male *Tie2-KLF11*–Tg mice and WT mice. There was no noticeable difference in body weight, blood pressure, plasma lipid profiles, MCP-1, and IL-6 between the 2 groups (Supplemental Figure 4, A–D). Meanwhile, elastase exposure did not destroy the endothelium integrity evidenced by immunofluorescence staining of CD31 and VE-cadherin (Supplemental Figure 4E). However, and consistent with the *Pcsk9*/AngII model, KLF11 overexpression in ECs significantly reduced elastase-induced AAA incidence (WT, 90% vs. *Tie2-KLF11*–Tg, 20%), abdominal aortic enlargement, elastin degradation, MMP9 expression in the endothelium, and leukocyte and macrophage accumulation, as well as the superoxide production in the aortic wall (Supplemental Figure 4, F–L). The above findings indicate that endothelial KLF11 protects against murine AAA development.

### Bone marrow–derived KLF11 does not affect AAA formation.

To exclude the effects of KLF11 loss or gain of function in blood cells (monocytes, lymphocytes, etc.) in AAA formation, bone marrow transplant (BMT) experiments were performed as described previously (20, 21). Briefly, the bone marrow cells isolated from *Klf11*^fl/fl^ and *Klf11*^ECKO^ mice were transplanted to irradiated WT mice, and the elastase-induced AAA model was performed on those mice (Supplemental Figure 5, A and B). Fourteen days after elastase exposure, the body weight, blood pressure, and plasma lipid profiles were comparable between the 2 groups of recipient mice (Supplemental Figure 5, C–E). The KLF11-deficient bone marrow cells did not impact AAA incidence, abdominal aortic enlargement, elastin degradation, and inflammatory cell accumulation in the aortic wall (Supplemental Figure 5, F–J). Those findings were further confirmed in another BMT experiment (Supplemental Figure 6). The WT and *Tie2-KLF11*–Tg mice transplanted with WT bone marrow were subjected to the elastase-induced AAA model. Two weeks after elastase exposure, there were no differences between the 2 groups in body weight, blood pressure, plasma lipid profiles, MCP-1, and IL-6 (Supplemental Figure 6, A–D). The AAA incidence, abdominal aortic dilation, elastin degradation, as well as the leukocyte and macrophage accumulation in the aortic wall were greatly decreased in the *Tie2-KLF11*–Tg mice transplanted with WT bone marrow compared with WT mice transplanted with WT bone marrow (Supplemental Figure 6, E–I). Collectively, these data indicate that increase in endothelial KLF11, rather than myeloid KLF11, is responsible for the KLF11 protective effects in AAA.

### KLF11 suppresses EC inflammatory activation.

Vascular inflammation is a fundamental element in the development of AAA (2, 3). ECs play an important role in vascular inflammation by expressing proinflammatory molecules (11). Our previous work demonstrated that KLF11 suppresses EC activation via NF-κB signaling (14). To unravel the protective mechanisms of KLF11 against endothelial dysfunction, we performed RNA-sequencing analysis of human aortic endothelial cells (HAECs) with *KLF11* knockdown using adenovirus-short hairpin RNA (Ad-sh*KLF11*), followed by gene set enrichment analysis (Supplemental Table 2). Of note, the pathways for inflammatory response (normalized enrichment score [NES] = 1.31, FDR *q* value = 0.161) and TNF-α signaling via NF-κB (NES = 1.35, FDR *q* value = 0.118) were enriched among the upregulated pathways in HAECs infected with Ad-sh*KLF11* (Supplemental Figure 7A), indicating that the inflammatory response is activated upon *KLF11* knockdown in ECs. The upregulation of proinflammatory genes (Supplemental Figure 7B and Supplemental Table 3), such as VCAM1 and E-selectin (encoded by *SELE*), was further validated through qPCR and Western blot. Consistently, *KLF11* knockdown significantly enhanced the expression of those proinflammatory genes under TNF-α stimulation (Supplemental Figure 7, C and D). Conversely, adenovirus-mediated *KLF11* overexpression (Ad-*KLF11*) significantly inhibited TNF-α–induced expression of those proinflammatory genes (Supplemental Figure 7, E and F). In addition, *KLF11* knockdown enhanced MCP-1 and IL-6 secretion from the TNF-α–stimulated HAECs, whereas *KLF11* overexpression decreased TNF-α–induced secretion of MCP-1 and IL-6 (Supplemental Figure 7, G and H). Consistent with the reduced secretion of MCP-1 and IL-6, the conditioned medium from *KLF11*-overexpressing HAECs inhibited macrophage migration (Supplemental Figure 7I). Additionally, leukocyte-EC adhesion assays showed that knockdown of *KLF11* in ECs using small interfering RNA (si*KLF11*) markedly increased TNF-α–mediated leukocyte adhesion to ECs, whereas *KLF11* overexpression in ECs significantly reduced the leukocyte-EC adhesion, concomitant with the changes of VCAM1, ICAM1, and E-selectin in ECs (Supplemental Figure 7, J and K). Taken together, our data demonstrate an inhibitory role of KLF11 on the migration and recruitment of inflammatory cells to ECs in vitro by modulating the expression and secretion of proinflammatory cytokines and adhesion molecules in ECs.

### KLF11 inhibits MMP9 expression in ECs.

**Figure 4:**
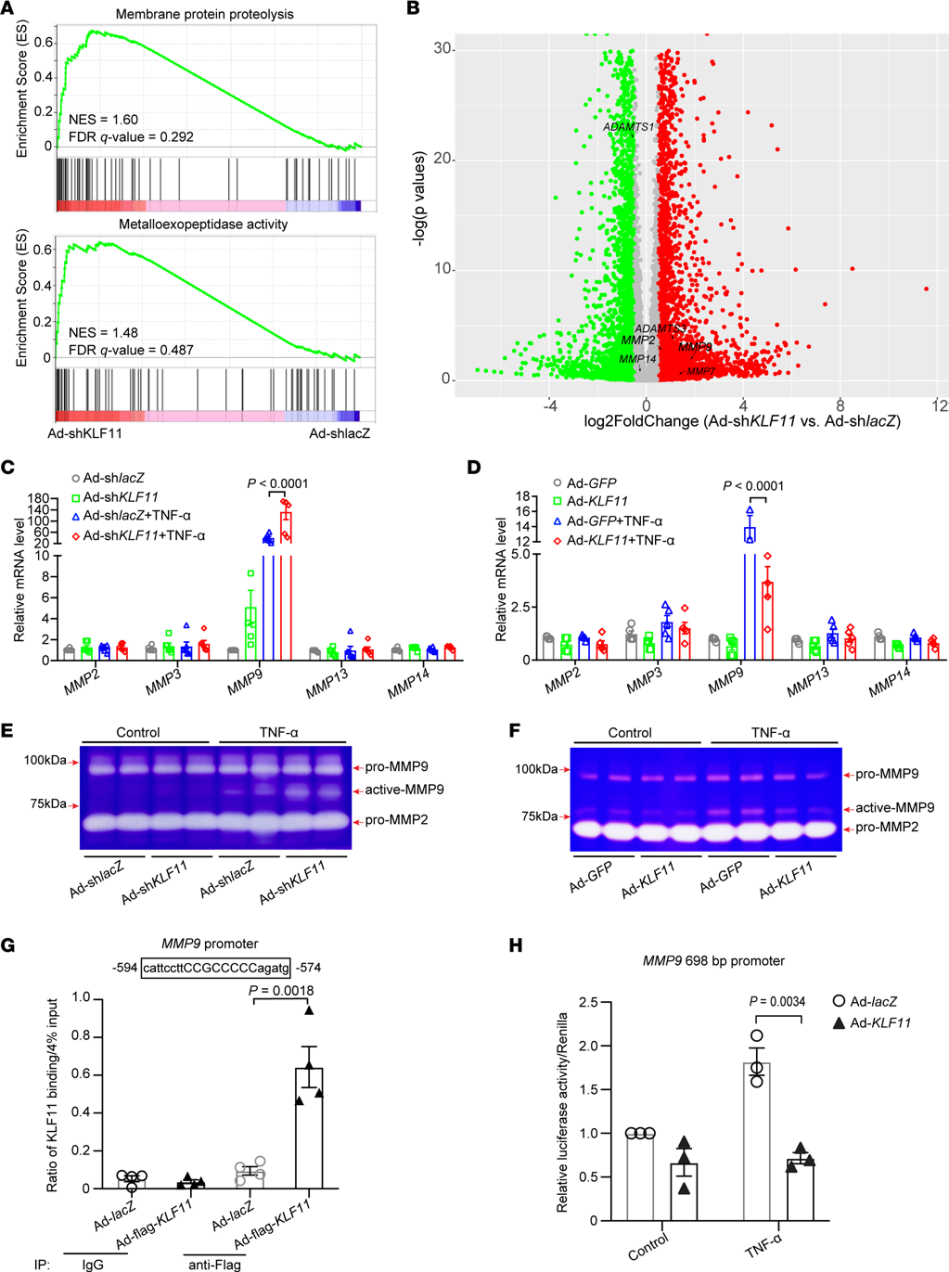
KLF11 inhibits MMP9 expression and activity in ECs. (**A** and **B**) Human aortic ECs (HAECs) were infected with adenovirus carrying shRNA for *KLF11* knockdown (Ad-sh*KLF11*, 10 MOI, *n* = 4) or lacZ (Ad-sh*lacZ*, *n* = 3) as control. After 48 hours, the total RNA was extracted for RNA sequencing. (**A**) The positive enrichment in the membrane protein proteolysis and metalloexopeptidase activity pathways is shown by gene set enrichment analysis plots (Ad-sh*KLF11* vs. Ad-sh*lacZ*). NES, normalized enrichment score. (**B**) The differentially expressed genes (Ad-sh*KLF11* vs. Ad-sh*lacZ*) are shown as volcano plots. Green dots, log_2_FoldChange < –0.5. Red dots, log_2_FoldChange > 0.5. Gray dots, –0.5 < log_2_FoldChange < 0.5. (**C**–**F**) HAECs were infected with Ad-sh*lacZ* or Ad-sh*KLF1*1 or Ad-*GFP* or Ad-*KLF11* (10 MOI). After 48 hours, they were treated with or without TNF-α (2 ng/mL) for 12 hours. (**C** and **D**) The mRNA levels of *MMP2*, *MMP3*, *MMP9*, *MMP13*, and *MMP14* were determined by qPCR. (**E** and **F**) Representative gelatin zymography for the activity of MMP2 and MMP9 in the conditioned medium. Samples from **E** and **F** were run on 8% and 10% SDS-PAGE gels with 0.1% gelatin, respectively. (**G**) HAECs were infected with Ad-*lacZ* or Ad-flag-*KLF11*. After 48 hours, they were stimulated with TNF-α (2 ng/mL) for 4 hours. Chromatin immunoprecipitation (ChIP) assay was performed using an antibody against flag or IgG. (**H**) Luciferase reporter assay in HAECs transfected with an *MMP9* promoter-driven luciferase reporter containing a KLF11 binding site and infected with Ad-*lacZ* or Ad-*KLF11*. After 48 hours, they were stimulated with TNF-α (2 ng/mL) for 4 hours. The luciferase activity was normalized against that of cotransfected *Renilla*. Data are presented as mean ± SEM. Two-way ANOVA followed by Holm-Sidak post hoc analysis (**C**, **D**, **G**, and **H**).

Increased expression of MMPs was observed in human and animal AAA tissues (22, 23). Consistently, the in vivo data showed that endothelial *Klf11* deficiency significantly enhanced MMP9 expression, whereas *KLF11* overexpression in ECs markedly reduced MMP9 expression in the endothelium from AAA models (Supplemental Figure 1H, Supplemental Figure 3G, and Supplemental Figure 4L). Moreover, gene set enrichment analysis (Supplemental Table 4) upon *KLF11* knockdown revealed that the pathways of membrane protein proteolysis (NES = 1.60, FDR *q* value = 0.292) and metalloexopeptidase activity (NES = 1.48, FDR *q* value = 0.487) were enriched in HAECs infected with Ad-sh*KLF11* (Figure 4A), accompanied by upregulation of multiple proteinases, such as *MMP2*, *MMP9*, and a disintegrin and metalloproteinase with thrombospondin motifs 3 (*ADAMTS3*) (Figure 4B and Supplemental Table 5). Next, we validated the expression of *MMP2*, *MMP9*, *MMP13*, and *MMP14* in HAECs using qPCR. *KLF11* knockdown enhanced while *KLF11* overexpression reduced TNF-α–induced expression of *MMP9* (Figure 4, C and D). Additionally, we noticed that inflammatory stimulus with TNF-α (2 ng/mL) increased the activation of MMP9 assessed by gelatin zymography in the EC-conditioned medium, while *KLF11* knockdown further enhanced TNF-α–induced activation of MMP9 (Figure 4E). In contrast, *KLF11* overexpression reduced the activation of MMP9 (Figure 4F). Next, chromatin immunoprecipitation (ChIP) assay performed in the HAECs demonstrated that KLF11 bound to the putative KLF binding site (–594 to –574) upstream of the *MMP9* transcription start site (Figure 4G). Furthermore, we generated a luciferase reporter construct containing the –693 to +5 region of the human *MMP9* promoter. In HAECs transfected with the *MMP9* reporter construct, *KLF11* overexpression significantly reduced the luciferase activity under TNF-α stimulation (Figure 4H). Multiple inflammatory genes and MMPs are direct targets of NF-κB signaling pathway (14, 24, 25). Our prior study demonstrated that KLF11 inhibits NF-κB signaling pathway via interaction with P65 (14). Consistently, we found that KLF11 overexpression reduced TNF-α–induced luciferase activity using a reporter containing NF-κB response elements (Supplemental Figure 8A). Moreover, administration of NF-κB pathway inhibitor (BAY11-7082, which inhibits the phosphorylation of IκBα) can abolish the effect of KLF11 knockdown on MMP9 expression in response to TNF-α in HAECs (Supplemental Figure 8B). Using ChIP assay, we found that KLF11 overexpression reduced the binding of P65 to the NF-κB binding site located at (–627 to –613) within *MMP9* promoter, which are close to the putative KLF11 binding sites (–594 to –574) (Supplemental Figure 8C). Collectively, our data indicate that KLF11 inhibition of MMP9 expression at the transcriptional level involves both NF-κB pathway–dependent and –independent mechanisms.

### KLF11 suppresses EC oxidative stress through inhibition of NOX2.

**Figure 5:**
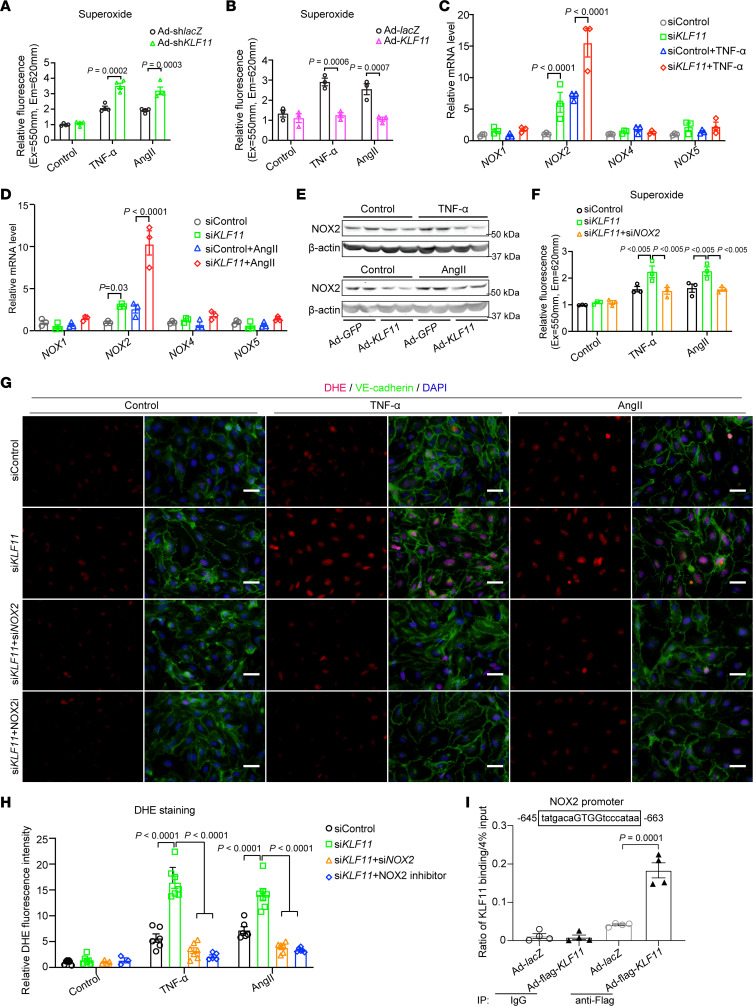
KLF11 attenuates ROS production through NOX2 suppression. (**A** and **B**) The production of superoxide was determined in HAECs. HAECs were infected with Ad-*lacZ* or Ad-*KLF11*, or Ad-sh*lacZ* or Ad-sh*KLF11* (10 MOI). After 48 hours, they were treated with TNF-α (2 ng/mL) or AngII (1 μM) for 2 hours in the presence of superoxide detection solution (fluorescent probes). (**C**–**E**) HAECs were infected with Ad-*GFP* or Ad-*KLF11* (10 MOI) or transfected with control siRNA (siControl) or *KLF11* siRNA (si*KLF11*, 20 μM). After 48 hours, they were stimulated with TNF-α (2 ng/mL) or AngII (1 μM) for 24 hours. (**C** and **D**) The mRNA levels of *NOX1*, *NOX2*, *NOX4*, and *NOX5* were determined by qPCR. (**E**) Western blot to determine the expression of NOX2 in HAECs. (**F**) HAECs were transfected with siControl, si*KLF11* or si*KLF11*+*NOX2* siRNA (si*NOX2*) (20 μM). After 48 hours, they were treated with TNF-α (2 ng/mL) or AngII (1 μM) for 2 hours in the presence of superoxide detection solution. (**G** and **H**) Representative DHE staining and quantification of superoxide production in HAECs. HAECs were transfected with siControl, si*KLF11*, or si*KLF11*+si*NOX2*. After 48 hours, they were pretreated with NOX2 inhibitor (NOX2i, GSK2795039, 1 μM) for 1 hour, then stimulated with TNF-α (2 ng/mL) or AngII (1 μM) for 2 hours, followed by DHE staining of superoxide (red) and immunofluorescence staining of VE-cadherin (green). Nuclei stained by DAPI are shown in blue. Scale bar: 20 μm. (**I**) HAECs were infected with Ad-*lacZ* or Ad-flag-*KLF11*. After 48 hours, they were stimulated with TNF-α (2 ng/mL) for 4 hours, followed by ChIP assay using an antibody against flag or IgG. Data are presented as mean ± SEM. Two-way ANOVA followed by Holm-Sidak post hoc analysis (**A**–**D**, **F**, **H**, and **I**).

Excessive ROS (e.g., superoxide, hydroxyl radical) has been implicated in the pathogenesis of AAA (26, 27). Consistently, superoxide was increased in the aortic walls of *Klf11*^ECKO^ mice in the AAA region, whereas KLF11 overexpression in ECs reduced superoxide production (Figure 2I and Supplemental Figure 4K). Interestingly, without stimuli, *KLF11* knockdown did not affect superoxide production in HAECs in vitro (Figure 5A). Multiple stimuli have been shown to induce ROS production in ECs, such as TNF-α and AngII (28, 29). Of note, *KLF11* knockdown significantly increased superoxide production in ECs under those stimuli (Figure 5A). Accordingly, *KLF11* overexpression decreased TNF-α– and AngII-induced superoxide production (Figure 5B). These results indicate that under pathological conditions, KLF11 deficiency increased endothelial superoxide production.

NOXs are the major source of superoxide production in human and animal vasculature (30, 31), and ECs can express 4 isoforms, i.e., NOX1, NOX2, NOX4, and NOX5 (28, 32). We found that *KLF11* knockdown markedly increased *NOX2* expression in HAECs under TNF-α and AngII treatment, without any significant effects on the expression of *NOX1*, *NOX4*, and *NOX5* (Supplemental Figure 9A and Figure 5, C and D). Conversely, *KLF11* overexpression reduced NOX2 expression in HAECs under the same stimuli (Figure 5E). Additionally, the increased superoxide production directly associated with KLF11 deficiency in the presence of TNF-α or AngII was abrogated by *NOX2* knockdown (si*NOX2*) (Figure 5F and Supplemental Figure 9B). Accordingly, DHE staining demonstrated that knockdown of *NOX2* or treatment with the NOX2 inhibitor (GSK2795039, NOX2i, 1 μM) significantly attenuated the increase in superoxide production associated with KLF11 downregulation (Figure 5, G and H). Bioinformatics analysis revealed a potential KLF binding site (–645 to –663 bp) upstream of the *NOX2* transcription start site. ChIP assay demonstrated that KLF11 bound to the promoter of *NOX2* to inhibit its expression (Figure 5I). Of note, knockdown of *KLF11* can upregulate *NOX2* expression both under basal conditions and in response to TNF-α. However, inhibition of NF-κB activation via BAY11-7082 could not abolish the effect of KLF11 on *NOX2* expression in the presence of TNF-α (Supplemental Figure 9C). Moreover, overexpression of *KLF11* did not affect the binding of P65 to the potential NF-κB binding site (–712 to –698 bp), close to the KLF11 binding site (–645 to –663 bp) within the *NOX2* promoter (Supplemental Figure 9D). These data suggest that KLF11 inhibits NOX2 expression at the transcriptional level mainly through directly binding to a consensus sequence within its promoter, particularly under basal conditions.

In human and animal AAAs, oxidative stress is a crucial trigger for vascular inflammation and MMP-dependent proteolysis (26, 27). Our data showed that enhanced expression of inflammatory molecules, such as *VCAM1*, *SELE*, *CCL2*, and *IL-6*, in HAECs with *KLF11* knockdown and stimulated with TNF-α, could be attenuated by *NOX2* knockdown (Supplemental Figure 9E). Recently, cyclophilin A (CypA) has emerged as a ROS-sensitive secreted cytokine in *Pcsk9*/AngII-induced aortic aneurysm and dissection (28). We found that *KLF11* knockdown significantly increased *CypA* expression in HAECs, and *CypA* expression was further enhanced upon TNF-α stimulation in combination with *KLF11* knockdown (Supplemental Figure 9F). Similarly, knockdown of *NOX2* completely abolished the increase in *CypA* expression induced by *KLF11* knockdown (Supplemental Figure 9F). Furthermore, knockdown of *NOX2* impaired the upregulation of *MMP9* in HAECs with *KLF11* knockdown (Supplemental Figure 9G).

Taken together, these results indicate that KLF11 inhibits NOX2 expression at the transcriptional level by directly binding to the *NOX2* promoter to preserve EC homeostasis while, under pathological conditions, KLF11 downregulation increases endothelial ROS production mediated, in part, through upregulation of NOX2, thus contributing to EC dysfunction.

### KLF11 expression in EC is critical for maintaining the SMC contractile phenotype.

**Figure 6:**
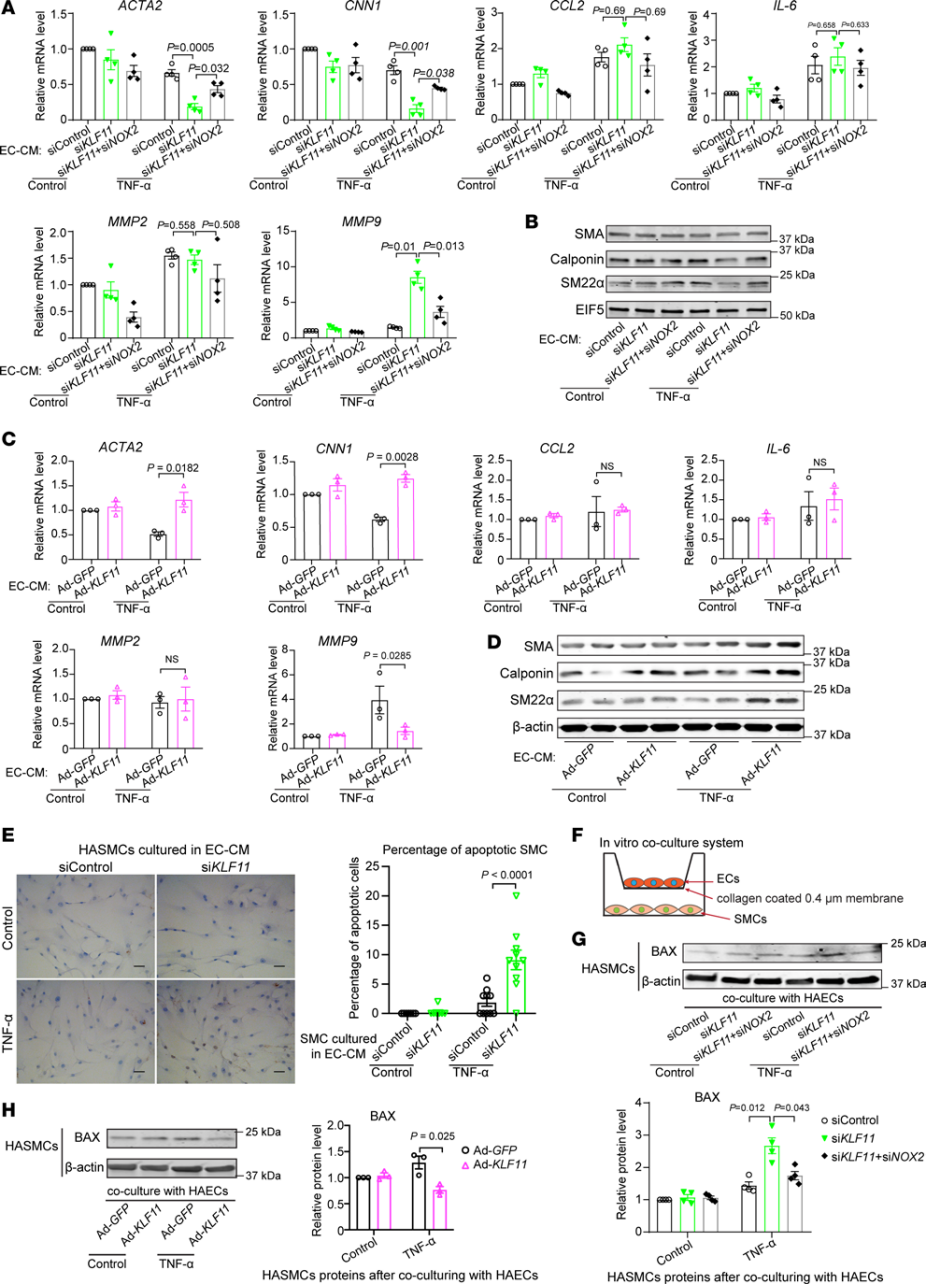
Coculture with KLF11-deficient ECs impairs SMC homeostasis. (**A**–**E**) Human aortic smooth muscle cells (HASMCs) were treated for 24 hours with the conditioned media from ECs (EC-CM) that had been transfected with siControl, si*KLF11*, or si*KLF11*+si*NOX2* (20 μM), or infected with Ad-*GFP* or Ad-*KLF11* (10 MOI) and subsequently stimulated for 1 hour with TNF-α (2 ng/mL) 48 hours after siRNA transfection or adenovirus infection and cultured in fresh opti-MEM for an additional 4 hours. (**A**–**D**) qPCR (**A** and **C**) and Western blot (**B** and **D**) to examine expression of SMC-specific contractile markers (smooth muscle α-actin [SMA], calponin, and smooth muscle 22–α [SM22α]), proinflammatory cytokines (MCP-1 and IL-6), and metalloproteinases (MMP2 and MMP9). (**E**) HASMCs were cultured in EC-CM for 48 hours, followed by immunostaining of TUNEL. Scale bar: 20 μm. (**F**–**H**) Schematics of the in vitro coculture system using a Transwell. HAECs (upper chamber) transfected with siControl, si*KLF11* (20 μM), or si*KLF11*+si*NOX2* or infected with Ad-*GFP* or Ad-*KLF11* (10 MOI) were cultured for 48 hours followed by TNF-α (2 ng/mL) stimulation for 1 hour separately from HASMCs, changed to fresh opti-MEM, and then cocultured with HASMCs (bottom) in fresh opti-MEM for 24 hours. The expression of BAX in HASMCs was assayed by Western blot (**G** and **H**). Data are mean ± SEM from 3 independent experiments. Two-way ANOVA followed by Holm-Sidak post hoc analysis (**A**, **C**, **E**, **G**, and **H**).

With the identification of increased SMC apoptosis within the aortic wall of *Klf11*^ECKO^ mice in the AAA model, we next sought to determine whether EC dysfunction induced by KLF11 deficiency was involved in regulating VSMC phenotypic transformation and apoptosis. First, we used the conditioned media from HAECs (EC-CM) with either *KLF11* knockdown or overexpression, subsequently treated with or without TNF-α (2 ng/mL), to culture human aortic SMCs (HASMCs) for 24 hours. The EC-CM from HAECs treated with vehicle did not affect SMC contractile genes’ expression (Figure 6, A–D). Of note, HASMCs grown in the EC-CM from HAECs with *KLF11* knockdown and treated with TNF-α displayed decreased expression of SMC contractile markers, such as SMA, calponin, and SM22α, and increased MMP9 expression, with no change in the expression of proinflammatory cytokines, such as MCP-1 and IL-6 (Figure 6, A and B). In contrast, the EC-CM from HAECs with *KLF11* overexpression and TNF-α stimulation significantly increased the expression of SMC contractile markers and reduced MMP9 expression relative to the EC-CM from Ad-*GFP*–infected HAECs in the same conditions (Figure 6, C and D).

Furthermore, the EC-CM from TNF-α–stimulated HAECs with *KLF11* knockdown significantly increased HASMC apoptosis, as assessed by TUNEL staining, compared with the EC-CM from siControl-transfected HAECs stimulated with TNF-α (Figure 6E). Additionally, we determined the expression of the apoptosis regulator BCL2-associated X protein (BAX) using a coculture system (Figure 6F). After 24 hours of coculture in fresh opti-MEM with HAECs that had been transfected with si*KLF11* 48 hours earlier, and stimulated with TNF-α (2 ng/mL) for 1 hour, HASMCs demonstrated increased BAX protein abundance in comparison with HASMCs cocultured with HAECs transfected with siControl and treated with TNF-α (Figure 6G). In contrast, in coculture with TNF-α–treated HAECs overexpressing *KLF11*, HASMCs showed decreased expression of BAX (Figure 6H). Notably, silencing of *NOX2* in ECs with *KLF11* knockdown and TNF-α stimulation could partially reverse the effect of KLF11 knockdown (Figure 6, A, B, and G). Taken together, our data indicate that KLF11 expression in ECs is required for maintaining the VSMC contractile phenotype and preventing VSMC apoptosis, 2 essential VSMC phenotypes involved in AAA.

## Discussion

AAA is a fatal arterial disease, which usually remains asymptomatic until rupture, at which stage results in 80% mortality (3). AAA is a complex pathophysiological process, and its underlying mechanisms remain incompletely understood. In this study, we demonstrated that endothelial KLF11 acts as a vasoprotective factor against AAA through multifaceted mechanisms that include reduction of inflammatory response, inhibition of MMP9 expression, and suppression of NOX2-mediated ROS production in ECs, as well as the preservation of VSMC contractile phenotype and viability.

Vascular inflammation and excessive inflammatory cell recruitment to the vascular wall are essential factors in the initiation and progression of AAA (2, 33). The recruitment of inflammatory cells is mediated primarily by EC activation, and the consequent increased expression of proinflammatory molecules, such as VCAM1, E-selectin, and MCP-1 (11, 34). In particular, TNF-α–induced NF-κB activation is one of the major pathways contributing to inflammation-mediated vascular injury (9, 35). Our previous study demonstrated that KLF11 reduced TNF-α–induced expression of adhesion molecules via inhibition of the NF-κB signaling pathway (14). However, the specific role of KLF11 in AAA remained unknown. In the current study, we uncovered that *Klf11* depletion in ECs enhanced leukocyte and macrophage accumulation in the aortic wall, both in *Pcsk9*/AngII- and elastase-induced AAA models, concomitant with increased expression of adhesion molecules and cytokines in ECs and increased monocyte adhesion in the presence of inflammatory stimuli. Furthermore, the protective role of EC-specific KLF11 in AAA was determined using BMT experiments and elastase-induced AAA model, to exclude a protective contribution of monocytic KLF11 to reduced AAA. Our study is the first to our knowledge to reveal a protective role of KLF11 in AAA formation through antiinflammatory effects on ECs and its homeostatic consequences on VSMCs.

The aortic ECM degradation by proteases, including MMPs (36), ADAMTSs (37), and cysteine proteases (36, 38), is a crucial step in the formation and progression of AAA, and these proteases are increased in the aortic wall from patients with AAA (37). However, the specific role of the endothelial MMPs in AAA progression remains poorly understood. Our observation that MMP9 expression was markedly and specifically increased in ECs from *Klf11*^ECKO^ mice in both AAA models used here indicates that endothelial KLF11 plays a crucial role in the regulation of MMP9 production by ECs. Moreover, we found that, under the proinflammatory stimuli, KLF11 not only inhibits MMP9 expression but also reduces the activation of MMP9 in ECs. Recently, endothelial MMP9 was described as a trigger of the inflammatory response in AAA (8). Moreover, ECM fragments proteolytically generated by MMPs also show bioactive properties and promote the recruitment of inflammatory cells, such as macrophages and T cells (39–41). Subsequently, the recruited inflammatory cells produce more inflammatory cytokines and proteases, which potentiate the inflammatory response and vascular injury. In this context, our finding identifies EC expression of MMP9 as a potentially critical mechanism that regulates vascular remodeling and inflammation, essential to AAA initiation and progression.

Oxidative stress is a leading cause of cardiovascular diseases in general and causes vessel damage due to increased production or impaired clearance of ROS (42, 43). Excessive ROS act as destructive agents affecting DNA and proteins, leading to inflammation and tissue injury (44, 45). In vascular ECs, the major sources of ROS are the NOXs (7, 42), and NOX2 is the main source of ROS production in response to pathological stimuli, such as TNF-α and AngII (28, 29, 46). Recently, EC-specific NOX2 overexpression and the resulting heightened ROS production were shown to increase the susceptibility to *Pcsk9*/AngII-induced aortic dissection (28), consistent with the increase of NOX2 abundance found in human aortic aneurysm tissues (27). However, relatively little is known about the mechanisms that regulate NOX2 expression in ECs. In this study, we show for the first time to our knowledge that deficiency of endothelial *KLF11* results in substantial upregulation of NOX2 and ROS production associated with endothelial *Klf11* knockout aggravation of the AAA pathogenesis. We show that *NOX2* knockdown reduced ROS production in ECs with *KLF11* knockdown and demonstrate that KLF11 is a crucial transcriptional repressor of NOX2 expression in ECs in response to AAA-relevant stimuli, thus establishing a direct association between KLF11 downregulation and enhanced NOX2-dependent ROS production in ECs. In addition, the expression of inflammatory molecules, including VCAM1, E-selectin, MCP-1, and IL-6, was partially inhibited after knockdown of *NOX2* in ECs with *KLF11* knockdown, suggesting that endothelial oxidative stress and inflammation concurrently increase the damage to the aortic wall. Although some studies support that oxidative stress contributes to the pathogenesis of AAA (26, 27) and antioxidant drugs, such as vitamin E, can reduce the size of AAA in preclinical experimental models (47), to date, no antioxidant therapy has proved effective at preventing AAA in humans. Our study points toward a causative role of endothelial NOX2 in the pathogenesis of AAA in experimental models, suggesting that this oxidase could be a molecular target for the treatment of AAA.

ECs also secrete a variety of substances that affect the neighboring cells. Recent studies show that ECs regulate the functions and phenotypes of VSMCs by releasing various bioactive agents, such as microRNA (48), nitric oxide (49), and enzymes (8). In this study, we showed that VSMCs stimulated with conditioned media or cocultured with ECs subjected to *KLF11* silencing had markedly lower expression of SMC-specific contractile markers, including SMA, calponin, and SM22α, but higher expression of MMP9 and enhanced apoptosis. In addition, oxidative stress and apoptosis in ECs and medial VSMCs were markedly enhanced in the aneurysmal aortas from *Klf11*^ECKO^ mice, suggesting that KLF11-dependent effects in ECs are essential for the interaction between ECs and VSMCs toward maintaining normal vascular physiology and structure in vivo. Accordingly, endothelial KLF11 deficiency leads to enhanced MMP9 production and apoptosis in VSMCs, further weakening the vascular wall and contributing to AAA formation. CypA, a ROS-dependent factor secreted from ECs, has been recently identified to mediate VSMC activation in AngII-induced aortic dissection (28). Consistently, *KLF11* knockdown also increased *CypA* expression in ECs and *NOX2* silencing could abolish this effect, suggesting that increased CypA in KLF11-deficient ECs may further contribute to inducing VSMC phenotypic changes. The conditioned media from ECs with *KLF11* knockdown and TNF-α stimulation potentially contain a great variety of factors, such as those identified here (namely, inflammatory cytokines, MMP9, CypA, and ROS), all of which can trigger or promote VSMC dedifferentiation and apoptosis. Hence, follow-up studies will focus on the systematic identification in the endothelial conditioned media of the gamut of KLF11-dependent secreted factors, which may act as intermediate molecules for the KLF11-dependent effects in the crosstalk between ECs and VSMCs and which could serve as targets of intervention for AAA.

In summary, we define for the first time to our knowledge EC-specific KLF11 as a novel protective factor against AAA operating by reducing endothelial inflammation and ROS production, improving EC function, and maintaining vascular homeostasis. These protective effects involve the newly identified KLF11 direct transcriptional inhibition of MMP9 and NOX2. Indeed, loss of KLF11 as AAA progresses directly allows NOX2-dependent ROS overproduction in ECs, causing endothelial activation, expression of adhesion molecules, secretion of chemokines, and production of ECM-degrading protease MMP9, which further amplify the vascular inflammation associated with AAA and other vascular diseases. These findings indicate that KLF11-dependent effects on ECs may define new targets for intervention in the treatment of AAA and cardiovascular disease at large.

## Methods

### Reagents.

KLF11 antibody (catalog X1710) was produced by Syd Labs. CD31 antibody (catalog DIA-310) was purchased from Dianova. CD45 antibody (550539) was purchased from BD Biosciences. Galectin 3 (Mac2) antibody (14-5301-85) was purchased from Thermo Fisher Scientific. The antibodies against MMP9 (ab38898), α–smooth muscle actin (ab119952), calponin (ab46794), and SM22α (ab103135) were from Abcam. The antibodies against VE-cadherin (sc-6458), VCAM1 (sc-13160), E-selectin (sc-14011), and EIF5 (sc-28309) were from Santa Cruz Biotechnology. NOX2 antibody (NBP2-67680) was purchased from Novus Biologicals. The antibodies against β-actin (catalog 4967), GAPDH (catalog 5174), Flag (DYKDDDDK) tag (catalog 14793), rabbit IgG (catalog 2729), and BAX (catalog 2772) were from Cell Signaling Technology (CST). Recombinant human TNF-α (catalog 210-TA) was from R&D Systems, Bio-Techne, and used at 2 ng/mL to stimulate endothelial cells. AngII (catalog H-1706) was purchased from Bachem and used at 1 μM to stimulate endothelial cells.

### Cell culture.

HAECs (CC-2535) from a 54-year-old male and HASMCs (CC-2571) from a 22-year-old male were purchased from Lonza. HAECs and HASMCs were cultured in EC growth media-2 (CC-3202, Lonza) and SMC growth medium-2 (C-22062, Promo Cell), respectively, at 37°C, 5% CO_2_, in a humidified cell culture incubator. Both HAECs and HASMCs were used from passages 4 to 8 in all experiments. The human monocyte cell line THP-1 was purchased from ATCC and grown in RPMI 1640 containing 10% FBS (Thermo Fisher Scientific) and 50 mg/mL of a penicillin/streptomycin solution.

### Animals and mouse AAA models.

The *Klf11*^fl/fl^ mice containing *loxP* sites flanking exon 3 of *Klf11* were generated at the Transgenic Animal Model Core facility at the University of Michigan from *Klf11* targeted embryonic stem cells purchased from University of California Davis. The B6.Cg-Tg(*Tek*-Cre)1Ywa/J *(Tie2-*Cre, stock 008863) mice were purchased from The Jackson Laboratory. The *Klf11*^ECKO^ mice on the C57BL/6 background were generated by crossbreeding *Klf11*^fl/fl^ mice with *Tie2*-Cre mice. The EC-selective *Klf11* transgenic mice (*Tie2-KLF11*–Tg) on the C57BL/6 background were generated at the Transgenic Animal Model Core facility at the University of Michigan by using a human *KLF11* ORF driven by a mouse *Tie2*-promoter. The C57BL/6J mice were purchased from The Jackson Laboratory.

The peri-adventitial elastase application–induced (elastase–induced) AAA model was performed as previously described (17, 50). Briefly, 10- to 12-week-old male mice were anesthetized by intraperitoneal injection of a mixture of ketamine (100 mg/kg) and xylazine (5 mg/kg). The infrarenal abdominal aorta was isolated and then surrounded with a sterile gauze, previously soaked with 30 μL of elastase (44 U/mL, MilliporeSigma, E1250). After 30 minutes of incubation, the gauze was removed, and the abdominal cavity was washed twice with sterile saline before suturing.

The *Pcsk9*/AngII-induced murine AAA model was performed as previously described (18, 19). In brief, 8- to 10-week-old male mice were injected intraperitoneally with 2 × 10^11^ genomic copies of adeno-associated virus (AAV, serotype 8) carrying a gain-of-function mutation of the mouse *Pcsk9* (AAV-*Pcsk9.D377Y*, Penn Vector Core at the University of Pennsylvania) and switched to a Western diet containing 0.2% cholesterol by weight (TD.88137, Envigo) to induce hypercholesterolemia. Two weeks after AAV injection, mice were subcutaneously implanted with osmotic mini-pumps (Alzet, model 2004) to infuse AngII (Bachem, H-1706) for 28 days at a releasing rate of 1500 ng/kg/min.

Fourteen days after elastase exposure or 28 days after AngII infusion, mice were euthanized, and blood was collected by ventricle puncture before perfusion with saline and 4% paraformaldehyde through the left ventricle to remove the remaining blood, followed by isolation of the aortas for ex vivo measurements. The maximal external diameters of the infrarenal abdominal aortas from the elastase-induced AAA model and suprarenal abdominal aortas from the *Pcsk9/*AngII-induced AAA model were determined. Diameters larger than 50% of those of the adjacent portion were considered as AAA (51). The mice with ruptures in the thoracic aorta were excluded from the calculation of incidence of *Pcsk9*/AngII-induced AAA. The systolic blood pressure was measured by a noninvasive tail-cuff method (Visitech BP-2000).

Plasma total cholesterol (TC) and triglyceride levels were measured by enzymatic kits (FUJIFILM Wako Diagnostics). The mice from the *Pcsk9*/AngII-induced AAA model with TC levels less than 250 mg/dL at endpoint were excluded from data analysis regardless of whether they had developed AAA. Serum MCP-1 and IL-6 levels were determined by ELISA at the Immunology Core at the University of Michigan.

### En face immunofluorescence staining.

Male C57BL/6J mice, 10 weeks old, were subjected to elastase- and *Pcsk9*/AngII-induced AAA models. After perfusion and fixation, the infrarenal abdominal aortas from mice at 0, 7, and 14 days after elastase exposure, and suprarenal abdominal aortas from mice with AngII infusion for 0, 7, and 14 days, were isolated and permeabilized for 10 minutes using a permeabilizing solution (0.1% Triton X-100 in PBS) with rocking at room temperature. After washing with PBS, the aortas were incubated in Tris-buffered saline with 2.5% Tween 20 (TTBS) with 10% normal donkey serum for 30 minutes with rocking at room temperature. The rabbit anti-KLF11 antibody (Syd Labs, X1710, 1:50 dilution) and goat anti–VE-cadherin (Santa Cruz Biotechnology, sc-6458, 1:50 dilution), or normal rabbit or goat IgG in TTBS containing 10% normal donkey serum, were incubated with the aortas overnight, with gentle rocking at 4°C. Alexa Fluor–conjugated secondary antibodies (Jackson ImmunoResearch Laboratories catalog numbers 711-585-152 and 805-605-180) were applied for 1 hour, with rocking at room temperature. After washing 3 times with TTBS, the aortas were longitudinally opened to expose the endothelium. The aortas were mounted on cover glasses using ProLong Gold Antifade Mountant with DAPI (Invitrogen, Thermo Fisher Scientific, P36935), with the endothelium facing down. Immunofluorescence images were captured with a Nikon A1 inverted confocal microscope.

### Aortic ECs from human patients with aortic aneurysm.

The human samples used in this study were from the Michigan Biorepository of the University of Michigan Cardiovascular Health Improvement Project (CHIP), in the Department of Surgery at the University of Michigan. Cardiac surgeons are routinely performing surgery to collect the tissues for the CHIP initiative with patient informed consent. All the human samples in CHIP are collected with specific approval from the Human Research Protection Program and Institutional Review Boards of the University of Michigan Medical School (Hum00077616). The aortic specimens were obtained from 5 patients with aortic aneurysm undergoing open surgical aortic repair, and 4 control samples were obtained during heart transplants (Supplemental Table 1). The aortic endothelial cells from fresh specimens were gently scraped from the aortic specimens and lysed in RIPA lysis buffer (Thermo Fisher Scientific, 89901) supplemented with the cOmplete EDTA-free protease inhibitor cocktail (Roche, 11873580001) and PhosSTOP phosphatase inhibitor (Roche, 4906845001), followed by Western blot.

### Isolation and culture of mouse pulmonary ECs.

Mouse pulmonary ECs were isolated by 2 rounds of cell sorting using CD31 and ICAM-2 antibody–coated magnetic beads as previously described (52, 53). In brief, aliquots of Dynabeads (Invitrogen, Thermo Fisher Scientific, 11305) were placed on DynaMag-Spin Magnet (Thermo Fisher Scientific, 12320D), washed with Dulbecco’s PBS (DPBS) (without Ca^2+^ and Mg^2+^) containing 0.1% BSA (0.1% BSA/DPBS) 3 times according to the manufacturer’s instructions, and incubated with CD31 antibody (BD Pharmingen, 553369) and then ICAM2 antibody (BD Pharmingen, 01800D), respectively, overnight with rotation at 4°C. The antibody-coated beads were washed 4 times with 0.1% BSA/DPBS. The 4-week-old mice were anesthetized using an intraperitoneal injection of ketamine (100 mg/kg) and xylazine (5 mg/kg), and the lung lobes were aseptically excised and placed in cold isolation buffer (DMEM supplemented with 20% heat-inactivated FBS, 20 mM HEPES, and 0.5 mg/mL penicillin/streptomycin). After removing the bronchi, the lung tissues were minced and digested in 2 mg/mL collagenase A (MilliporeSigma, 10103586001) for 30 minutes with gentle rocking at 37°C. The digested tissue suspension was further dissociated by aspirating the suspension up and down more than 12 times with a syringe fitted with a 14G needle. The cell suspension was then pipetted through a 70 μm strainer and centrifuged at 400*g* for 5 minutes at 4°C. The cell pellets were resuspended with 0.1% BSA/DPBS and incubated with the anti-CD31–coated beads (15 μL beads/mL cell suspension) for 10 minutes with gentle rotating at room temperature. The beads and sorted ECs were washed with isolation buffer 5 times and cultured in growth medium (DMEM supplemented with 1 mol/L HEPES, 10 mg/mL heparin, 50 mg/mL penicillin/streptomycin, 20% FBS, 1 mM sodium pyruvate, 1× nonessential amino acid solution [Gibco, Thermo Fisher Scientific, 11114-050], and 20 μM l-glutamine [Gibco, Thermo Fisher Scientific, 25030-081]) on a collagen I–coated T75 flask. When the cells grew to 90% confluence, the cells were detached with 0.25% trypsin-EDTA and subjected to the second round of sorting with anti-ICAM2–coated beads following the same steps as those for the first sorting. More than 80% of the sorted cells were ECs, as verified by vWF and VE-cadherin immunostaining. The cells were used for experiments after the culture reached about 80% confluence.

### Histology.

The suprarenal abdominal aortas (from the diaphragm to the right renal artery) from the AngII-induced AAA model and the infrarenal abdominal aortas from the elastase-induced AAA model were excised. The aortas were embedded in paraffin at the In-Vivo Animal Core from the University of Michigan. The serial sections (5 μm thick, 200 μm apart) were deparaffinized, rehydrated, and stained with a Verhoeff-van Gieson staining kit (Electron Microscopy Sciences) for elastin assessment according to the manufacturer’s instructions. Based on the previous report (19, 51), elastin degradation was graded as: 1, <25% degradation; 2, 25% to 50% degradation; 3, 50% to 75% degradation; or 4, >75% degradation.

For immunostaining, the rehydrated sections were boiled in citrate buffer at pH 6.0 (Invitrogen, Thermo Fisher Scientific, 00-5000) for epitope retrieval for 15 minutes. After blocking with 5% donkey serum in PBS for 1 hour at room temperature, the sections were incubated with primary antibody against CD45 (BD Biosciences, 550539,1:50), Mac2 (Thermo Fisher Scientific, 14-5301-85, 1:100), MMP9 (Abcam, ab38898, 1:100), or VE-cadherin (Santa Cruz Biotechnology, sc-6458, 1:50) at 4°C overnight. After washing with PBS, the sections were incubated with Alexa Fluor–conjugated secondary antibody (Jackson ImmunoResearch Laboratories) for 1 hour at room temperature. The negative controls were sections incubated with species-matched IgG. Slides were mounted with ProLong Gold Antifade Mountant with DAPI (Invitrogen, Thermo Fisher Scientific, P36935) before image collection with an Olympus DP73 microscope. Quantification analysis of the numbers of infiltrated leukocytes (CD45^+^) and macrophages (Mac2^+^) in the aortic wall and the expression of MMP9 in the endothelium were performed with ImageJ software (NIH).

### Dihydroethidium staining.

The DHE staining of mouse aorta sections and HAECs was conducted as previously described (54). Briefly, the suprarenal abdominal aortas (AngII-induced AAA) and infrarenal abdominal aortas (elastase-induced AAA) were embedded in OCT. Serial cryosections (7 μm thick, 200 μm apart) were rehydrated in PBS (pH 7.5). The aorta sections were immediately incubated with fresh DHE staining solution (dissolved in PBS at a concentration of 5 μM) for 30 minutes at 37°C protected from light. After washing 3 times with PBS, the slides were mounted with ProLong Gold Antifade Mountant with DAPI (Invitrogen, Thermo Fisher Scientific, P36935), and images were captured with an Olympus DP73 microscope. Quantitative analysis of the DHE fluorescence within the aortic wall was performed with ImageJ software.

HAECs were transfected with siControl, si*KLF11*, or si*KLF11*+si*NOX2* for 48 hours, or treated with *NOX2* inhibitor for 1 hour, then stimulated with TNF-α (2 ng/mL) or AngII (1 μM) for 2 hours or their corresponding vehicle controls. Next, the cells were subjected to DHE staining. Image collection and quantification analysis of the DHE fluorescence in the HAECs were performed as described above.

### Bone marrow transplantation.

The protocol for syngeneic BMT was performed as previously described (20, 21). In brief, the recipient mice at 6 weeks of age were given 13 Gy (1300 rad) whole-body irradiation from a Cesium-137 gamma source. The donor bone marrow cells were isolated from the femurs of WT mice and resuspended at a concentration of 2 × 10^7^ cells/mL in serum-free RPMI (RPMI 1640 + 20 mM HEPES + 50 mg/mL of a penicillin/streptomycin solution). A mixture of 5 × 10^6^ bone marrow cells (total volume = 250 μL) was injected by tail vein into each of the recipient mice 4 hours after irradiation. Two weeks after BMT, the elastase-induced AAA model was performed on the recipient mice.

### Apoptosis assay.

The DeadEnd Fluorometric TUNEL system (Promega, G3250) was used to detect the vascular cell apoptosis within the aortic wall according to the manufacturer’s protocol. Briefly, the sectioned aortic tissue (7 μm thick) was permeabilized with Proteinase K solution (20 μg/mL) for 10 minutes at room temperature, then incubated with equilibration buffer for 10 minutes at room temperature. The terminal deoxynucleotidyl transferase (TdT) reaction mixture was added to the aortic tissues for 60 minutes with incubation at 37°C, followed by 2× SSC buffer to stop the reaction. Slides were mounted with ProLong Gold Antifade Mountant with DAPI (Invitrogen, Thermo Fisher Scientific, P36935), and the green fluorescence of apoptotic cells within the aortic wall was captured with an Olympus DP73 microscope. The ApopTag Peroxidase in Situ Apoptosis Detection Kit (MilliporeSigma, S7100) was used to detect the apoptosis of cultured HAECs according to the manufacturer’s instructions. Briefly, HAECs were fixed in 1% paraformaldehyde for 10 minutes at room temperature and a precooled mixture of ethanol and acetic acid (2:1 by volume) for 5 minutes at –20°C sequentially, followed by 3% hydrogen peroxide for 5 minutes at room temperature to quench endogenous peroxidase. After incubation in equilibration buffer, HAECs were treated with working strength TdT enzyme solution for 60 minutes at 37°C, followed by working strength Stop/Wash buffer to stop the reaction. Then anti-digoxigenin conjugate and peroxidase substrate were sequentially incubated with HAECs, and the apoptotic cells were recorded by microscopy.

### siRNA transfection.

HAECs were transfected with 30 nM si*KLF11* (Thermo Fisher Scientific, s13158), si*NOX2* (Thermo Fisher Scientific, s531915), or SilencerSelect Negative Control siRNA (siControl, Thermo Fisher Scientific, 4390843) using Lipofectamine RNAiMAX Reagent (Invitrogen, Thermo Fisher Scientific, 13778150) according to the manufacturer’s instructions.

### Total RNA isolation and quantitative real-time PCR analysis.

Total RNA from HAECs or HASMCs was extracted using RNeasy Mini Kit (QIAGEN, 74106) according to the manufacturer’s instructions. SuperScriptIII First-Strand Synthesis System (Thermo Fisher Scientific, 18080051) and random primers were used to reverse-transcribe RNA into cDNA. Gene expression was quantified by Real-Time PCR Detection System (Bio-Rad) using iQ SYBR Green Supermix (Bio-Rad, 1708882). The gene expression level was normalized to the internal control GAPDH. The primer sequences used are listed in Supplemental Table 6.

### RNA sequencing.

Total RNA was extracted from HAECs 48 hours after infection with Ad-sh*lacZ* (*n* = 3) or Ad-sh*KLF11* (*n* = 4), using RNeasy Mini Kit (QIAGEN, 74106), and then treated with RNase-free DNase I (QIAGEN, 79254) according to the manufacturer’s instructions. RNA library preparation and sequencing were performed by the Advanced Genomics Core at the University of Michigan. In brief, RNA quality was determined by BioAnalyzer (Agilent) before sequencing. The RNA library was prepared with NEBNext Ultra RNA Library Prep Kit (New England Biolabs), and 51 bp reads paired-end sequencing was performed on a HiSeq 6000 platform (Illumina). A total of 266 million reads were generated, with an average of 38 million reads for each sample. RNA-Seq read mapping was performed as described previously (55). Briefly, FastQC (Babraham Bioinformatics) was used for quality control of the sequencing reads from each sample. Gene expression quantification was performed using Salmon v 0.14.0 (56), with human cDNA sequences of GRCh38 (Ensembl database) as reference. The differential expression analysis was performed with the DeSeq2 package in R (57). Gene set enrichment analysis (58, 59) was performed to interpret gene expression profiles of Ad-sh*lacZ*– and Ad-sh*KLF11*–infected HAECs. Genes were mapped to the HALLMARK and GO gene set in the Molecular Signatures Database for pathway analysis. The RNA-Seq raw data and processed data described in the paper have been deposited in the National Center for Biotechnology Information’s Gene Expression Omnibus database (https://www.ncbi.nlm.nih.gov/geo/) under the accession code GSE152468.

### Protein extraction and Western blot.

Cells were lysed in RIPA lysis buffer (Thermo Fisher Scientific, 89901) supplemented with the cOmplete EDTA-free protease inhibitor cocktail (Roche, 11873580001) and PhosSTOP phosphatase inhibitor (Roche, 4906845001). Protein extracts were resolved in 10% SDS-PAGE gels and transferred to nitrocellulose membranes (Bio-Rad, 1620115). Membranes were blocked in TBST containing 5% fat-free milk for 1 hour at room temperature and incubated with primary antibodies at 4°C overnight. After washing 3 times with 1× TBST, membranes were incubated with secondary antibody (1:10,000 dilution, LI-COR Biosciences catalog numbers 926-32213, 926-68072, and 926-32214) for 1 hour at room temperature. After 3 washes with 1× TBST, bands were scanned using Odyssey Imaging System (LI-COR Biosciences) and quantified with the LI-COR Image Studio Software.

### Leukocyte-EC assay.

The THP-1 and HAEC adhesion assay was performed as described before (14). In brief, THP-1 cells were infected with Ad-*GFP*. HAECs were infected with Ad-*lacZ* or Ad-*KLF11*, or transfected with siControl or si*KLF11*. After 48 hours, HAECs were treated with TNF-α (2 ng/mL) or vehicle control for 4 hours and subsequently incubated for 30 minutes with Ad-*GFP*–infected THP-1 cells. The unbound THP-1 cells were removed by washing with PBS 3 times. The adhered cells were fixed with 4% paraformaldehyde and photographed by fluorescence microscopy, and numbers were calculated from 9 random fields per well using ImageJ software.

### EC-CM and gelatin zymography.

A total of 2 × 10^4^/cm^2^ HAECs seeded in 6-well culture plates in 10% serum containing medium were infected with Ad-*GFP*, Ad-*KLF11*, Ad-sh*lacZ*, or Ad-sh*KLF11* (10 MOI). After 48 hours, HAECs were treated with human recombinant TNF-α (2 ng/mL) for 1 hour, then cultured in fresh opti-MEM (Gibco, Thermo Fisher Scientific). After 4 hours, media were collected as EC-CM and transferred to HASMCs for 24 hours.

Gelatin zymography was performed as previously described (51). Briefly, equal volumes of EC-CM were electrophoresed on SDS-PAGE gels containing 1 mg/mL gelatin (MilliporeSigma, G8150). Gels were washed 3 times in 2.5% Triton X-100 and incubated in zymography buffer (50 mM Tris-HCl, 150 mM NaCl, 10 mM CaCl_2_, and 0.05% sodium azide) for 48 hours at 37°C. Gels were stained/destained with eStain L1 Protein Staining kit (Genscript, M00549) using the eStain L1 Protein Staining device (Genscript).

### Chromatin immunoprecipitation assay.

ChIP assays were performed using the SimpleChIP Enzymatic Chromatin IP Kit (Magnetic Beads) (CST, 9003S) according to the manufacturer’s protocol. In brief, HAECs were infected with Ad-*lacZ* or Ad-flag-*KLF11*. After 48 hours, HAECs were incubated with 1% formaldehyde for 10 minutes at room temperature, and the cross-linking was stopped by 0.1% glycine at room temperature for 5 minutes. The nuclei pellets were digested with Micrococcal Nuclease at 37°C for 20 minutes, followed by sonication (Branson Sonifier SLPe, 10 seconds of 35% amplification, 3 times). After centrifugation at 9,400*g* for 10 minutes at 4°C, the chromatin was incubated with an anti-flag antibody (1:100, CST, 14793S) or normal rabbit IgG (CST, 2729) at 4°C overnight with gentle rotation. The DNA/protein complexes were immunoprecipitated by ChIP-grade protein G magnetic beads with rotation for 2 hours at 4°C, followed by 3 washes in low-salt buffer, 1 wash in high-salt buffer, and elution at 65°C for 30 minutes. The eluted DNA-protein complexes were reversed with proteinase K at 65°C for 2 hours. The DNA was purified and then amplified by real-time qPCR with the following primers targeted to the Mmp9 promoter (–626/–535): forward primer: 5′-AGTGGAATTCCCCAGCCTT-3′ and reverse primer: 5*′*-CCTGACAGCCTTCTTTGACTCA-3*′*. Nox2 promoter (–720/–620), forward primer: 5*′*-TCAAAGTGCTGGGATTACAGGC-3*′* and reverse primer: 5′-GCTTTGGCCAATGATGATGAACCAC-3′.

### Cellular ROS/superoxide detection assay.

The production of superoxide and ROS in HAECs was measured using the Cellular ROS/Superoxide Detection Assay Kit (Abcam, ab139476) following the manufacturer’s protocol. Briefly, HAECs were infected with Ad-sh*KLF11* or Ad-*KLF11* or transfected with si*KLF11* or si*NOX2* and, after 48 hours, seeded in 96-well black-wall/clear-bottom plates at a density of 1.5 × 10^4^ cells/wall. Negative control samples were pretreated with a ROS inhibitor (N-acetyl-l-cysteine) 30 minutes prior to stimulation. HAECs were stimulated with TNF-α (2 ng/mL) or AngII (1 μM), and 100 μL/well of ROS/superoxide detection solution was simultaneously added to cells for 2 hours at 37°C in the dark. The fluorescence was detected using a fluorescence microplate reader at excitation/emission 490/520 nm (for ROS) and excitation/emission 520/600 nm (for superoxide).

### Statistics.

Statistical analyses were performed using GraphPad Prism 8.0 software (GraphPad Software) or RStudio (for RNA-Seq). Unless indicated otherwise, data are presented as mean ± SEM. All data were evaluated for normality and variance. For normally distributed data, Student’s 2-tailed *t* test was used to compare the difference between 2 groups, and 1-way ANOVA followed by Tukey’s post hoc analysis or 2-way ANOVA followed by Holm-Sidak post hoc analysis were used for comparison among 3 or more groups. For data that were not normally distributed, nonparametric tests, including Mann-Whitney *U* test, χ^2^ test, or Mantel-Cox test (survival percentage), were used to compare 2 groups. *P* < 0.05 was considered statistically significant. All results are representative of at least 3 independent experiments.

### Study approval.

All animal studies and experimental procedures were performed according to the protocols approved by the IACUC at the University of Michigan. The human samples used in this study were obtained from the CHIP core in the Department of Cardiac Surgery at the University of Michigan with the Institutional Review Board approval (Hum0077616) from the Human Research Protection Program and Institutional Review Boards of the University of Michigan Medical School.

## Author contributions

GZ, ZC, YZ, and JZ performed the experiments and results analysis. GZ and JZ wrote the article. YG, HL, WL, OR, JS, HW, TZ, YF, LC, BY, and MTGB provided technical support and contributed to the discussion of the project. MTGB did the critical editing of the article. YEC and JZ designed the research and discussed the results.

## Supplementary Material

Supplemental data

## Version 1 01/28/2021

In-Press Preview

## Version 2 03/08/2021

Electronic publication
