# Supplementary material for: KLF11 protects against abdominal aortic aneurysm through inhibition of endothelial cell dysfunction
Source: JCI Insight. 2021 Mar 8;6(5):e141673. doi: 10.1172/jci.insight.141673 (PMC8021107; doi:10.1172/jci.insight.141673)
Supplement: Supplemental data [file jciinsight-6-141673-s093.pdf]

Supplemental Data

KLF11 protects against abdominal aortic aneurysm through inhibition of endothelial cell dysfunction

Guizhen Zhao[1], Ziyi Chang[1,2], Yang Zhao[1], Yanhong Guo[1], Haocheng Lu[1], Wenying Liang[1], Oren Rom[1], Huilun Wang[1], Jinjian Sun[1,3], Tianqing Zhu[1], Yanbo Fan[1,4], Lin Chang[1], Bo Yang[5], Minerva T. Garcia-Barrio[1], Y. Eugene Chen[1,5] and Jifeng Zhang[1]

Affiliations: : [1]Department of Internal Medicine, University of Michigan Medical Center, Ann Arbor, MI 48109; [2]Department of Pathology, China-Japan Friendship Hospital, Beijing 100029, China ; [3]Department of Cardiovascular Medicine, the Second Xiangya Hospital, Central South University, Changsha, Hunan 410011, China; [4]Department of Cancer Biology, College of Medicine, University of Cincinnati, Cincinnati, OH 45267; [5]Department of Cardiac Surgery, University of Michigan Medical Center, Ann Arbor, MI 48109.

Corresponding Authors:

Jifeng Zhang, PhD, Department of Internal Medicine, University of Michigan Medical Center, NCRC Bldg26-357S. 2800 Plymouth Rd, Ann Arbor, MI 48109, Email: jifengz@umich.edu

Y. Eugene Chen, MD, PhD, Department of Internal Medicine, University of Michigan Medical Center, NCRC Bldg26-361S. 2800 Plymouth Rd, Ann Arbor, MI 48109, Email: echenum@umich.edu

Supplementary Figures

1

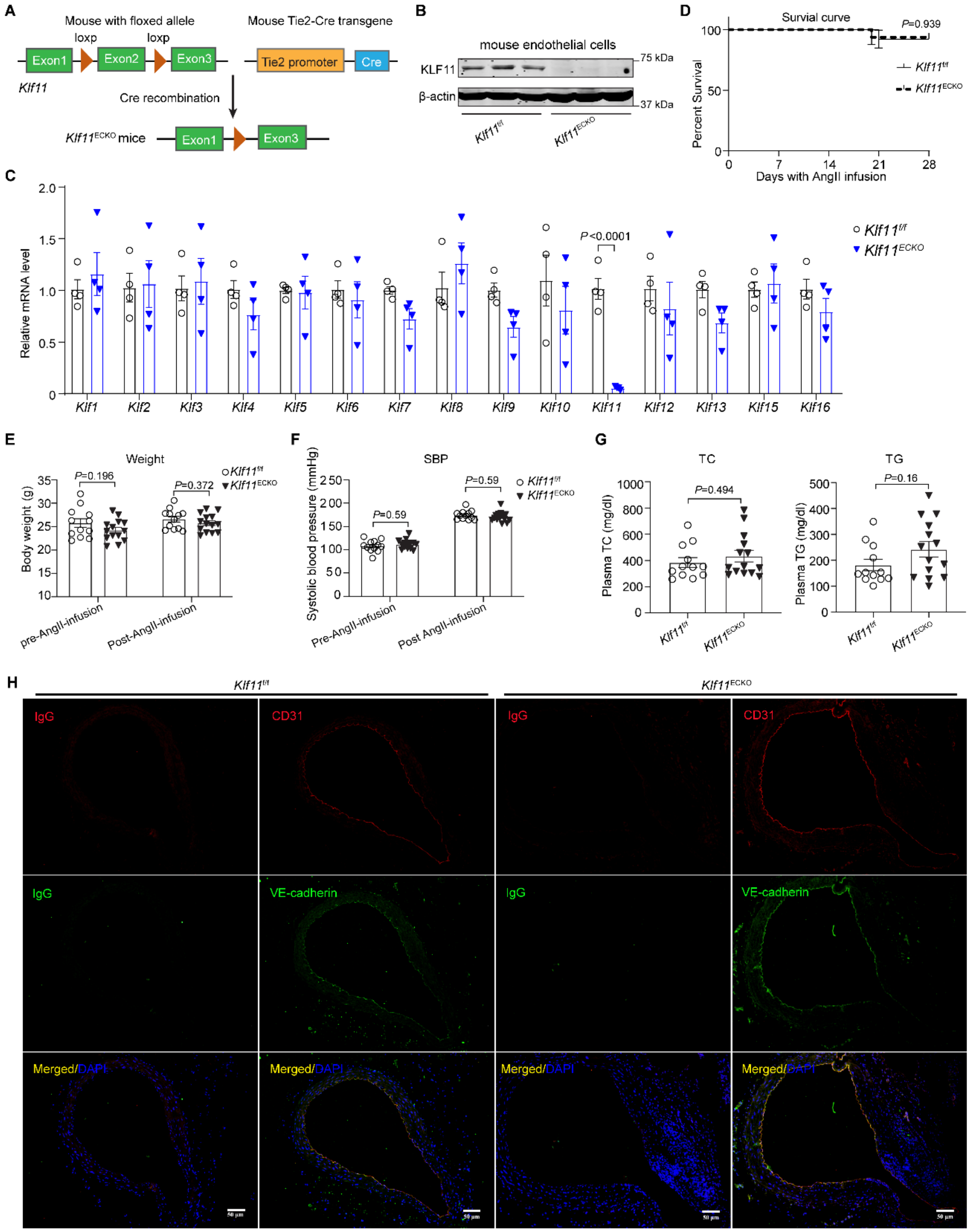
A
Mouse with floxed allele
loxp
loxp
Exon1
Exon2
Exon3
Klf11
Mouse Tie2-Cre transgene
Tie2 promoter
Cre
Cre recombination
Klf11ECKO mice
Exon1
Exon3
B
mouse endothelial cells
KLF11
β-actin
75 kDa
37 kDa
Klf11f/f
Klf11ECKO
D
Survial curve
P=0.939
Percent Survival
Klf11f/f
Klf11ECKO
Days with AngII infusion
C
Relative mRNA level
P<0.0001
Klf11f/f
Klf11ECKO
Klf1
Klf2
Klf3
Klf4
Klf5
Klf6
Klf7
Klf8
Klf9
Klf10
Klf11
Klf12
Klf13
Klf15
Klf16
E
Weight
P=0.196
P=0.372
Body weight (g)
pre-AngII-infusion
Post-AngII-infusion
F
SBP
P=0.59
P=0.59
Systolic blood pressure (mmHg)
Pre-AngII-infusion
Post AngII-infusion
G
TC
P=0.494
Plasma TC (mg/dl)
TG
P=0.16
Plasma TG (mg/dl)
H
Klf11f/f
Klf11ECKO
IgG
CD31
IgG
VE-cadherin
Merged/DAPI

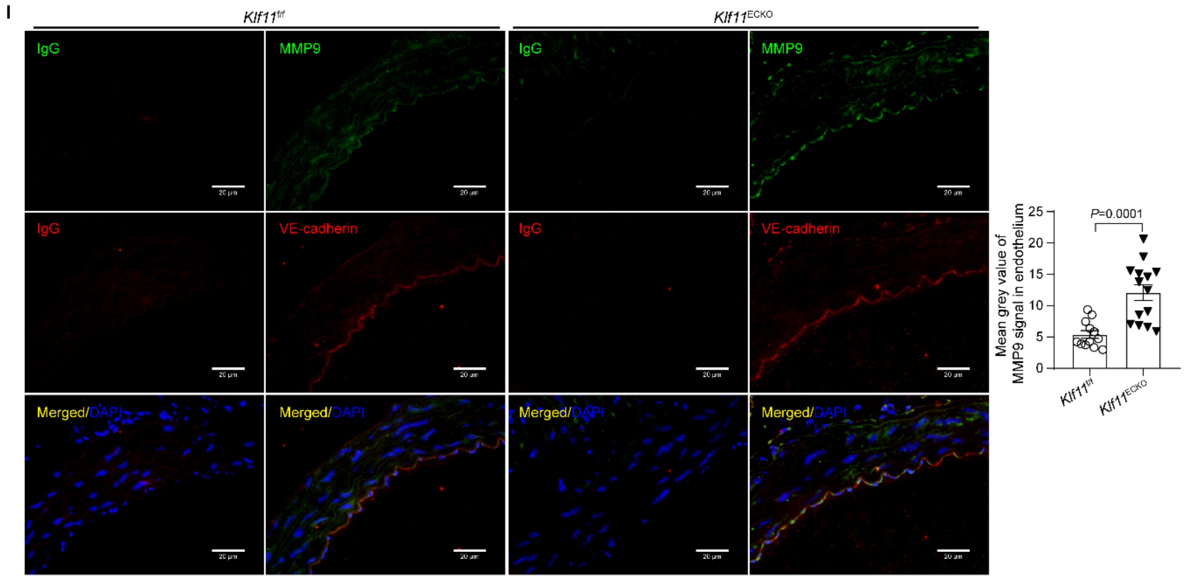

Supplementary Fig. 1: *Pcsk9*/AngII-induced AAA model in EC-specific *Klf11* knockout (*Klf11*^{ECKO}) and *Klf11* floxed control (*Klf11*$^{\mathrm{f/f}}$) mice. A. Schematic diagram of EC-specific *Klf11* knockout mice generated by cross-breeding floxed-*Klf11* (*Klf11*$^{\mathrm{f/f}}$) mice with the *Tie2*-Cre mice. **B**. Western blot analysis of KLF11 expression in the mouse pulmonary endothelial cells isolated from *Klf11*$^{\mathrm{f/f}}$ and *Klf11*^{ECKO} mice. **C.** qPCR to examine the expression of the *Klf* family members in mouse pulmonary endothelial cells isolated from *Klf11*$^{\mathrm{f/f}}$ and *Klf11*^{ECKO} mice. **D-I,** Ten-week-old *Klf11*$^{\mathrm{f/f}}$ (n=13) and *Klf11*^{ECKO} (n=15) male mice were given AAV-*Pcsk9* (i.p.) and Western diet to induce hypercholesterolemia followed by AngII (1500 ng/kg/min) infusion for 4 weeks. **D**. Kaplan-Meier survival curve of *Klf11*$^{\mathrm{f/f}}$ (n=13) and *Klf11*^{ECKO} (n=15) mice with AngII infusion for 4 weeks. **E-G**. Body weight (**E**), systolic blood pressure (**F**), and plasma total cholesterol (TC) and triglycerides (TG) (**G**) in *Klf11*$^{\mathrm{f/f}}$ (n=12) and *Klf11*^{ECKO} (n=14) mice. **H**. Representative immunofluorescence staining of CD31 (red) and VE-cadherin (green) in the suprarenal abdominal aortas from AngII-infused *Klf11*$^{\mathrm{f/f}}$ and *Klf11*^{ECKO} mice. Nuclei stained by DAPI are blue. Scale bar=50 μm. **I**. Representative immunofluorescence staining of MMP9 (green) and VE-cadherin (red) and quantification of MMP9 expression in the endothelium of the suprarenal abdominal aortas from AngII-infused *Klf11*$^{\mathrm{f/f}}$ (n=12) and *Klf11*^{ECKO} (n=14) mice.

Nuclei stained by DAPI are blue. Scale bar=20 µm. Data are presented as mean±SEM. Student's *t*-test for C and I, Mantel-Cox test for D*,* two-way ANOVA followed by Holm-Sidak post hoc analysis for E-F, Mann-Whitney test for G.

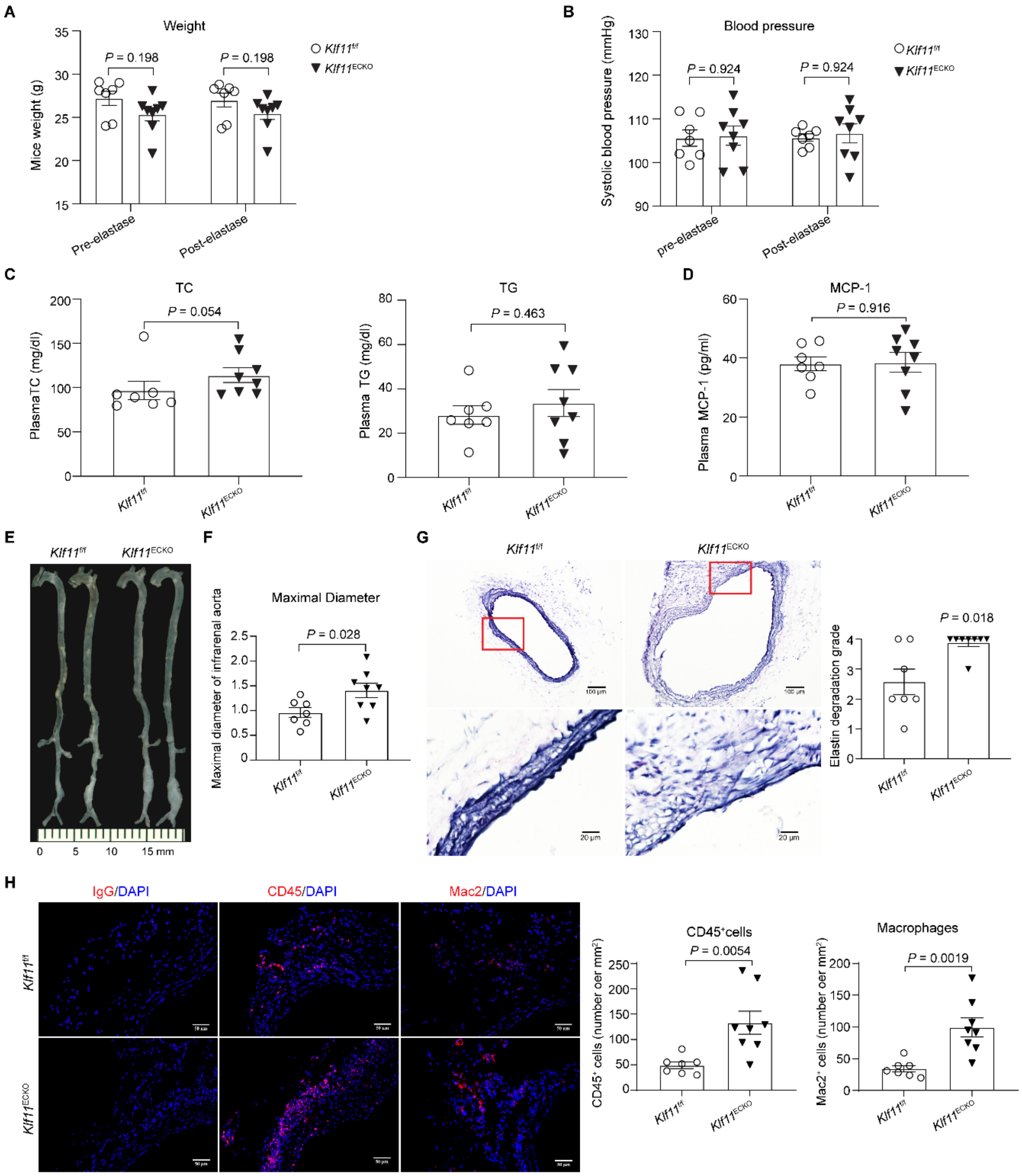

Supplementary Fig. 2: Endothelial cell-specific KLF11 depletion aggravates elastase-induced. **A-G**. Eight to twelve-week-old *Klf11*^{ECKO} (n=8) and *Klf11*$^{f/f}$ (n=7) male mice were subjected to elastase-induced AAA by the treatment of the infrarenal aortas with 30 µL elastase for 30 min as described in the Supplemental Methods and euthanized 14 days after elastase incubation. **A-D**. Body weight (**A**), systolic blood pressure (**B**), plasma total cholesterol and

triglycerides (**C**), and MCP-1 (**D**) in *Klf11*$^{f/f}$ and *Klf11*^{ECKO} mice. **E**. Representative morphology of aortas from *Klf11*$^{f/f}$ and *Klf11*^{ECKO} mice 14 days after elastase exposure. **F**. Quantification of maximal diameters of infrarenal abdominal aortas (IAAs) from *Klf11*$^{f/f}$ and *Klf11*^{ECKO} mice. **G**. Representative Verhoeff-Van Gieson (VVG) staining and quantification of elastin degradation in the IAAs from *Klf11*$^{f/f}$ and *Klf11*^{ECKO} mice. Scale bar=100 μm for whole aortic sections; scale bar=20 μm for magnified areas. **H**. Representative immunofluorescence staining and quantification of leukocyte ($CD45^+$) and macrophage ($Mac2^+$) infiltration in the adventitia of IAAs from *Klf11*$^{f/f}$ and *Klf11*^{ECKO} mice. Nuclei stained by DAPI are blue. Scale bar=50 μm. Data are presented as mean±SEM. Two-way ANOVA followed by Holm-Sidak post hoc analysis for A-B, Mann-Whitney test for C and G, Student's *t*-test for D, F and H.

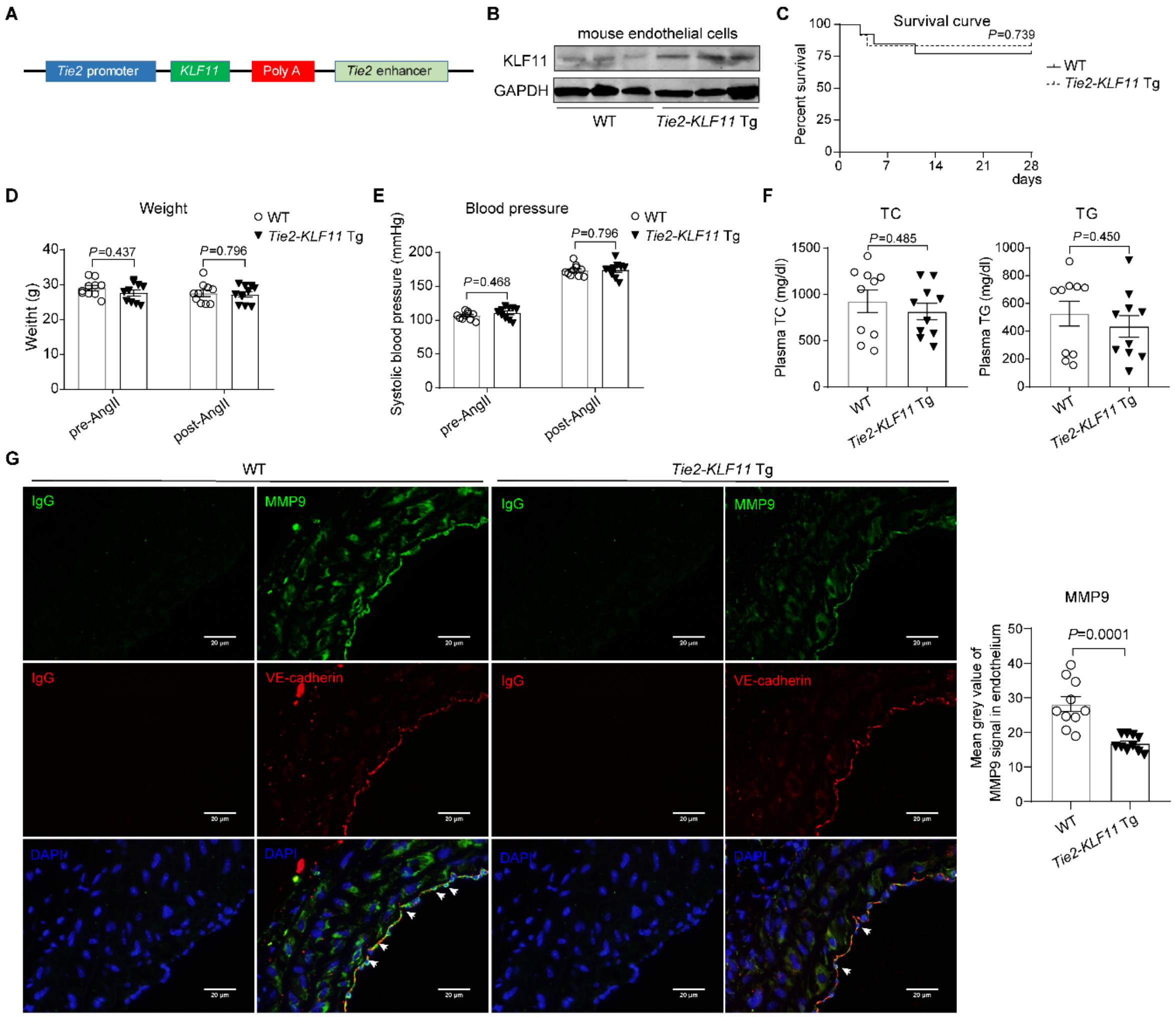

Supplementary Fig. 3: Endothelial cell-selective overexpression of KLF11 attenuates *Pcsk9*/AngII-induced AAA. **A**. Schematic diagram of the EC-selective *KLF11* transgene structure. **B**. Western blot analysis of KLF11 expression in the mouse endothelial cells isolated from WT and *Tie2-KLF11* Tg mice. **C-G**. *Pcsk9/AngII*-induced AAA model was performed on 8-week-old male EC-selective *KLF11* transgenic mice (*Tie2-KLF11* Tg, n=12) and littermate control mice (WT, n=13). **C**. Kaplan-Meier survival curve of WT (n=13) and *Tie2-KLF11* Tg (n=12) mice with AngII infusion for 4 weeks. **D-F**. Body weight (**D**), systolic blood pressure (**E**), plasma total cholesterol and triglycerides levels (**F**) in WT (n=10) and *Tie2-KLF11* Tg (n=10) mice. **G**. Representative immunofluorescence staining of MMP9 (green) and VE-cadherin (red) and quantification of MMP9 expression in the endothelium of the suprarenal abdominal aortas

from AngII-infused WT (n=10) and *Tie2-KLF11* Tg (n=10) mice. Nuclei stained with DAPI are blue. Scale bar=20 μm. Data are presented as mean±SEM. Mantel-Cox test for C*,* two-way ANOVA followed by Holm-Sidak post hoc analysis for D-E, Student's *t*-test for F-G.

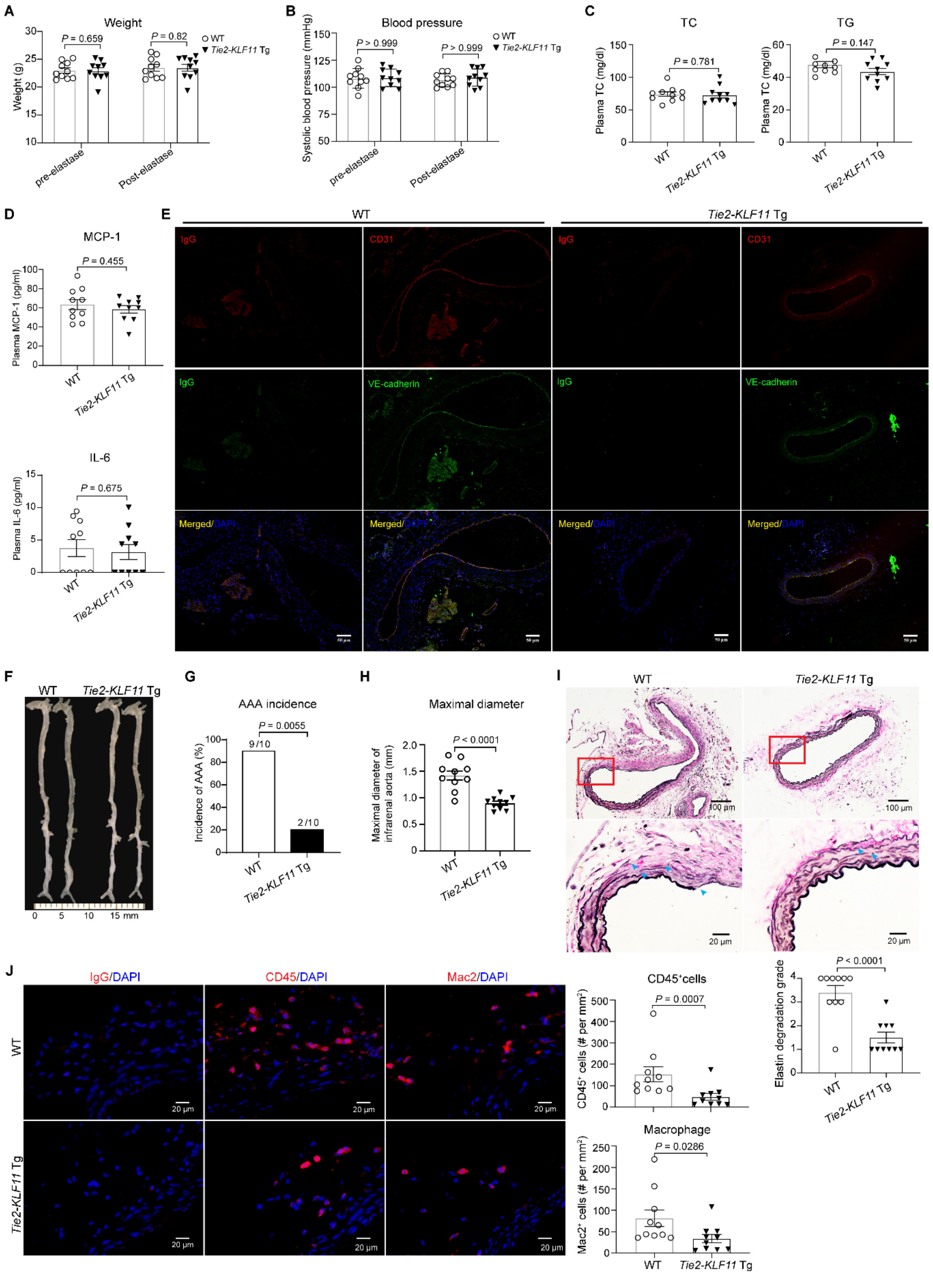
A
Weight
P = 0.659
P = 0.82
WT
Tie2-KLF11 Tg
Weight (g)
pre-elastase
Post-elastase
B
Blood pressure
P > 0.999
P > 0.999
Systolic blood pressure (mmHg)
pre-elastase
Post-elastase
C
TC
TG
P = 0.781
P = 0.147
Plasma TC (mg/dl)
WT
Tie2-KLF11 Tg
D
MCP-1
P = 0.455
Plasma MCP-1 (pg/ml)
IL-6
P = 0.675
Plasma IL-6 (pg/ml)
E
WT
Tie2-KLF11 Tg
IgG
CD31
VE-cadherin
Merged/DAPI
F
WT
Tie2-KLF11 Tg
0 5 10 15 mm
G
AAA incidence
P = 0.0055
9/10
2/10
Incidence of AAA (%)
H
Maximal diameter
P < 0.0001
Maximal diameter of infrarenal aorta (mm)
I
WT
Tie2-KLF11 Tg
100 µm
20 µm
J
IgG/DAPI
CD45/DAPI
Mac2/DAPI
WT
Tie2-KLF11 Tg
20 µm
CD45⁺cells
P = 0.0007
CD45⁺ cells (# per mm²)
Macrophage
P = 0.0286
Mac2⁺ cells (# per mm²)
P < 0.0001
Elastin degradation grade

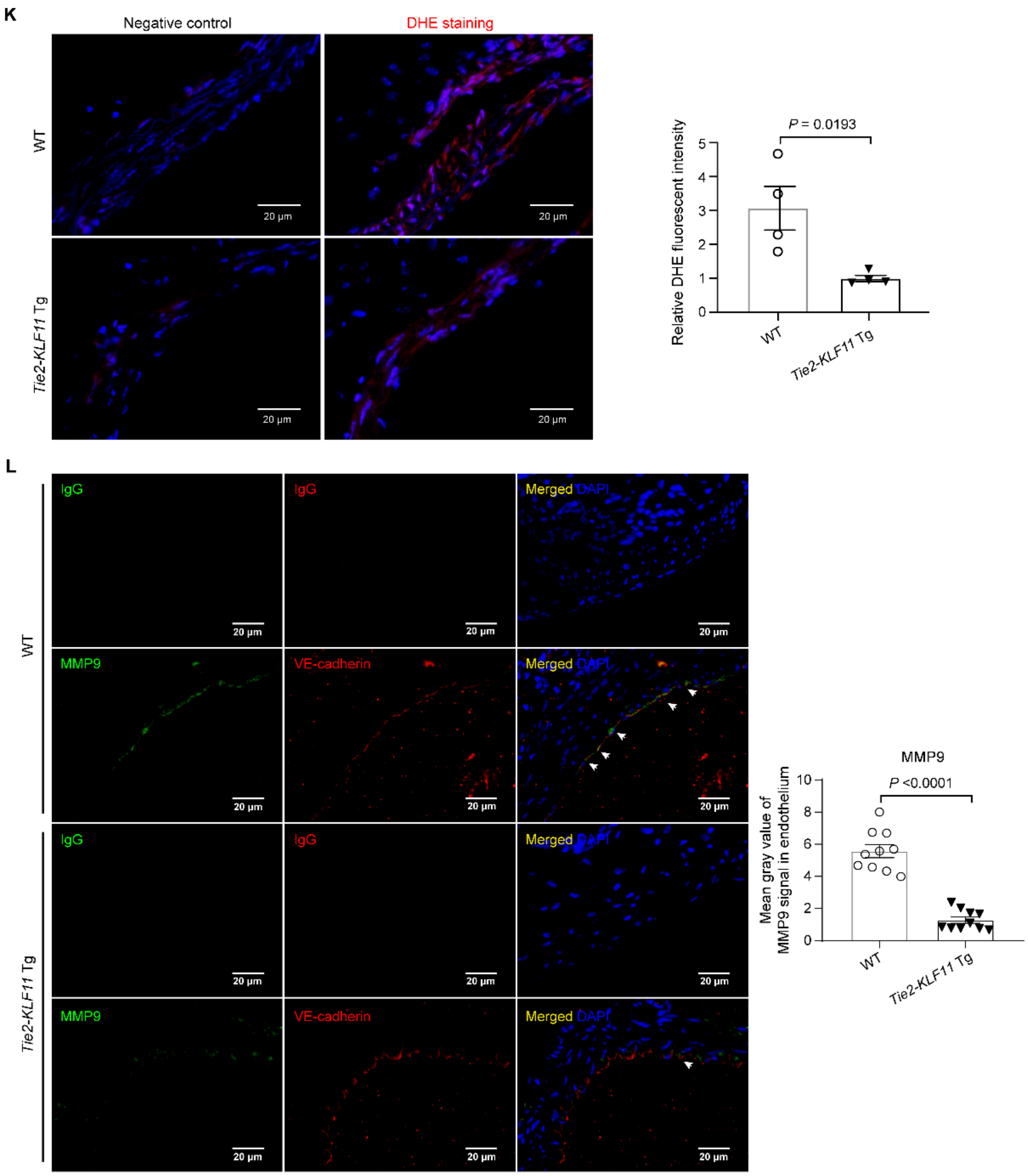

Supplementary Fig. 4: Endothelial cell-selective overexpression of KLF11 attenuates elastase-induced AAA. Ten-week-old WT (n=10) and *Tie2-KLF11* Tg (n=10) male mice were subjected to elastase-induced AAA. **A-D**. Body weight (**A**), systolic blood pressure (**B**), plasma total cholesterol and triglycerides (**C**) and MCP-1, IL-6 (**D**) in WT and Tie2-*KLF11* Tg mice. **E**. Representative immunofluorescence staining of CD31 (red) and VE-cadherin (green) in the IAAs from WT and *Tie2-KLF11* Tg mice. Nuclei stained by DAPI are blue. scale bar=50 μm. **F**. Representative morphology of aortas from WT and *Tie2-KLF11* Tg mice 14 days after elastase

incubation. **G**. Incidence of AAA. **H**. Maximal diameters of IAAs. **I**. Representative VVG staining and quantification of elastin degradation in IAAs from WT and *Tie2-KLF11* Tg mice (n=10/group). Scale bar=100 μm for whole aortic sections; Scale bar=20 μm for magnified areas. **J**. Representative immunofluorescence staining and quantification of leukocyte ($CD45^+$) and macrophage ($Mac2^+$) infiltration in the aortic wall of IAAs from WT and *Tie2-KLF11* Tg mice (n=10/group). Scale bar=20 μm. **K**. Representative DHE staining (red) and quantification of ROS production in the aortic wall of IAAs from WT and *Tie2-KLF11* Tg mice (n=4/group). Scale bar=20 μm. **L**. Representative immunofluorescence staining of MMP9 (green) and VE-cadherin (red) and quantification of MMP9 expression in the endothelium of the IAAs from WT and *Tie2-KLF11* Tg mice (n=10/group). Nuclei stained by DAPI are blue. Scale bar=20 μm. Data are presented as mean±SEM. Two-way ANOVA followed by Holm-Sidak post hoc analysis for A-B, Student's *t*-test for C, H, J and L, Mann-Whitney test for D and K, Chi-square test for G.

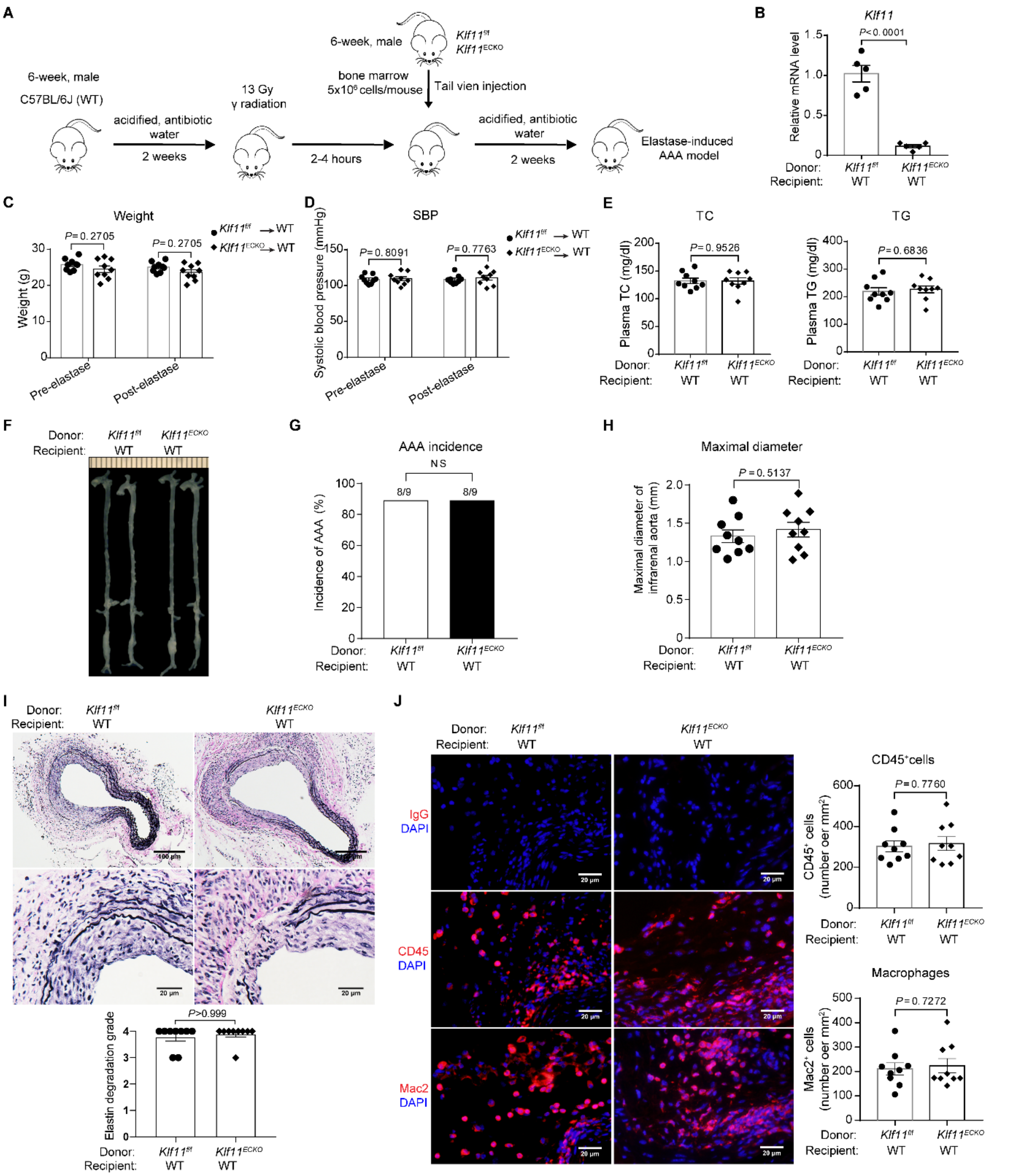

Supplementary Fig. 5: Bone marrow-derived KLF11 does not impact elastase-induced AAA formation. A. Schematics of the experimental design. Briefly, recipient WT mice received whole-body irradiation and were reconstituted with 5X10^6 bone marrow cells from donor *Klf11*$^{f/f}$ or *Klf11*^{ECKO} mice. The elastase-induced AAA model was performed on the recipient mice 2

weeks after bone marrow transplantation. **B**. qPCR analysis of *Klf11* expression in the bone marrow cells isolated from the recipient mice. **C-E**. Body weight (**C**), systolic blood pressure (**D**), plasma total cholesterol and triglycerides (**E**) in the recipient mice. **F**. Representative morphology of aortas from recipient mice transplanted with *Klf11*$^{f/f}$ or *Klf11*^{ECKO} bone marrow cells and sacrificed 14 days after elastase exposure. **G**. Incidence of AAA in the recipient mice. **H**. Quantification of maximal infrarenal abdominal aortic diameters from the recipient mice. **I**. Representative Verhoeff-Van Gieson staining and quantification of elastin degradation in IAAs from recipient mice. Scale bar=100 μm for whole aortic sections; Scale bar=20 μm for magnified areas. **J**. Representative immunofluorescence staining and quantification of leukocyte (CD45$^+$) and macrophage (Mac2$^+$) infiltration in the adventitia of IAAs from the recipient mice. Nuclei stained with DAPI are blue. Scale bar=20 μm. Data are presented as mean±SEM. Student's *t*-test for B, E, H and J, Two-way ANOVA followed by Holm-Sidak post hoc analysis for C-D, Chi-squared test for G, Mann-Whitney test for I.

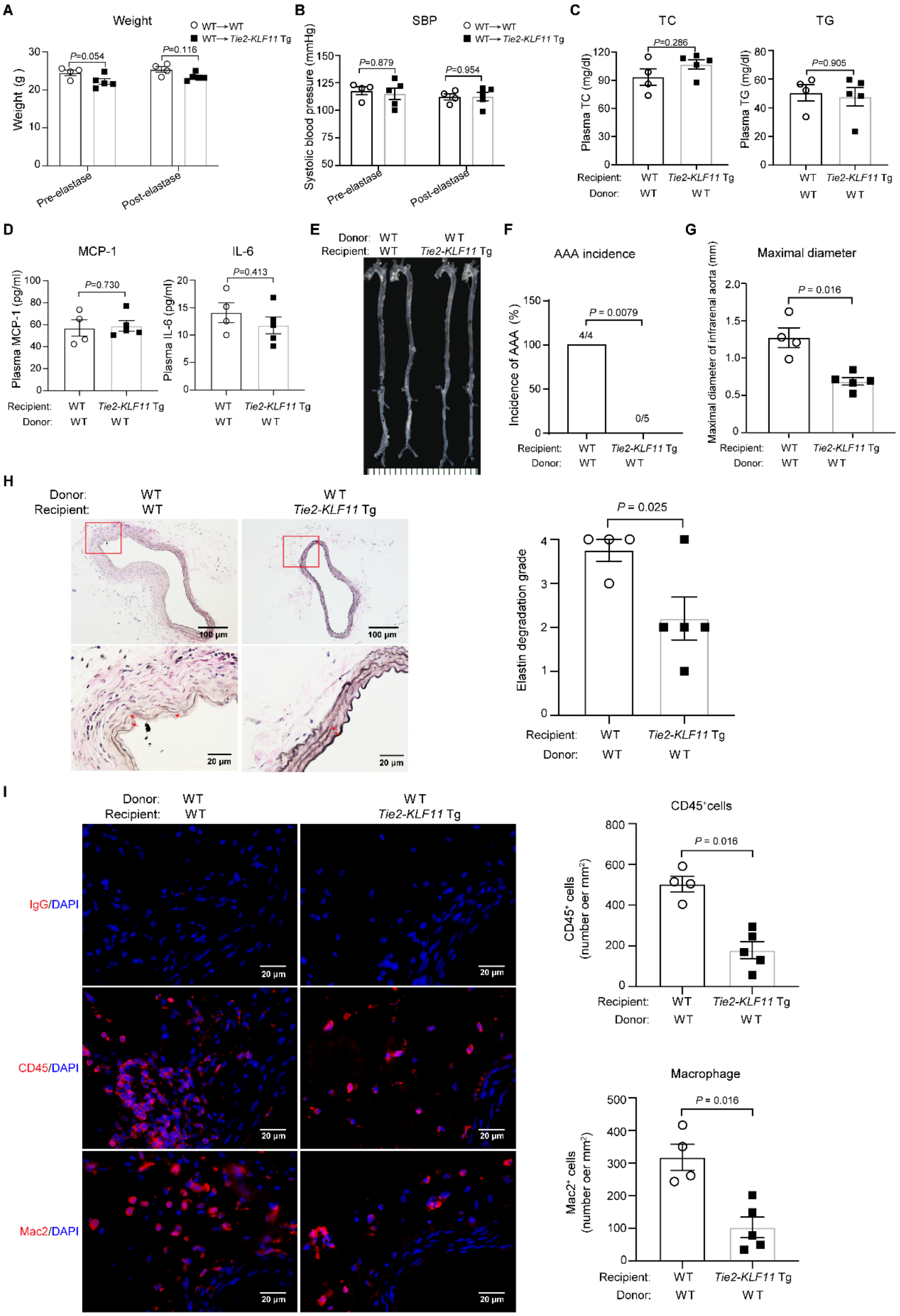
A
Weight
WT→WT
WT→ Tie2-KLF11 Tg
Weight (g)
P=0.054
P=0.116
Pre-elastase
Post-elastase
B
SBP
Systolic blood pressure (mmHg)
P=0.879
P=0.954
C
TC
TG
Plasma TC (mg/dl)
Plasma TG (mg/dl)
P=0.286
P=0.905
Recipient: WT Tie2-KLF11 Tg
Donor: WT WT
D
MCP-1
IL-6
Plasma MCP-1 (pg/ml)
Plasma IL-6 (pg/ml)
P=0.730
P=0.413
E
Donor: WT WT
Recipient: WT Tie2-KLF11 Tg
F
AAA incidence
Incidence of AAA (%)
P = 0.0079
4/4
0/5
G
Maximal diameter
Maximal diameter of infrarenal aorta (mm)
P = 0.016
H
Donor: WT WT
Recipient: WT Tie2-KLF11 Tg
100 μm
20 μm
Elastin degradation grade
P = 0.025
I
Donor: WT WT
Recipient: WT Tie2-KLF11 Tg
IgG/DAPI
CD45/DAPI
Mac2/DAPI
CD45+cells
CD45+ cells (number oer mm2)
P = 0.016
Macrophage
Mac2+ cells (number oer mm2)
P = 0.016

1

Supplementary Fig. 6: Bone marrow-derived KLF11 does not impact elastase-induced AAA formation. **A-I**. The recipient WT and *Tie2-KLF11* Tg mice received whole-body irradiation and were reconstituted with 5X10^6 donor WT bone marrow cells. Elastase-induced AAA model was performed on the recipient mice 2 weeks after bone marrow transplantation. **A-D**. Body weight (**A**), systolic blood pressure (**B**), plasma total cholesterol and triglycerides (**C**), and MCP-1, IL-6 (**D**) in recipient WT and *Tie2-KLF11* Tg mice. **E**. Representative morphology of aortas from the recipient WT and *Tie2*-KLF11 Tg mice transplanted with WT bone marrow cells and sacrificed 14 days after elastase exposure. **F**. Incidence of AAA in the recipient WT and *Tie2-KLF11* Tg mice. **G**. Quantification of maximal infrarenal abdominal aortic diameters from recipient WT and *Tie2-KLF11* Tg mice. **H**. Representative Verhoeff-Van Gieson staining and quantification of elastin degradation in infrarenal abdominal aortas from the recipient WT and *Tie2-KLF11* Tg mice. Scale bar=100 µm for whole aortic sections; Scale bar=20 µm for magnified areas. **I**. Representative immunofluorescence staining and quantification of leukocyte ($CD45^+$) and macrophage ($Mac2^+$) infiltration in the adventitia of IAAs from the recipient WT and *Tie2-KLF11* Tg mice. Nuclei stained with DAPI are blue. Scale bar=20 µm. Data are presented as mean±SEM. Two-way ANOVA followed by Holm-Sidak post hoc analysis for A-B, Mann-Whitney test for C-D, and G-I, Chi-squared test for F.

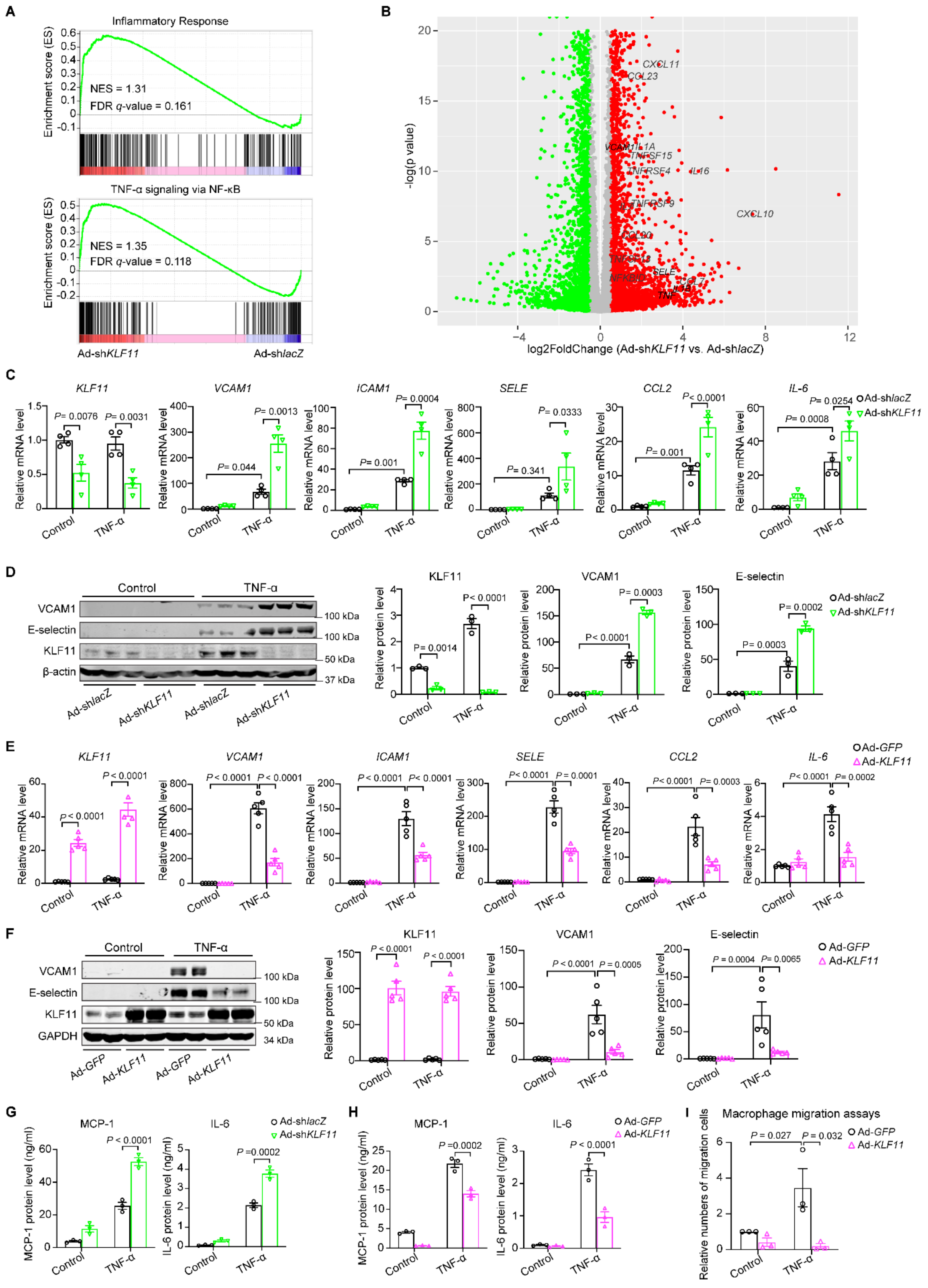
A
Inflammatory Response
Enrichment score (ES)
NES = 1.31
FDR q-value = 0.161
TNF-α signaling via NF-κB
NES = 1.35
FDR q-value = 0.118
Ad-shKLF11
Ad-shlacZ
B
-log(p value)
log2FoldChange (Ad-shKLF11 vs. Ad-shlacZ)
CXCL11
CCL23
CXCL10
IL16
C
KLF11
VCAM1
ICAM1
SELE
CCL2
IL-6
Relative mRNA level
Control
TNF-α
Ad-shlacZ
Ad-shKLF11
D
Control
TNF-α
VCAM1
E-selectin
KLF11
β-actin
100 kDa
50 kDa
37 kDa
Relative protein level
E
Ad-GFP
Ad-KLF11
F
GAPDH
34 kDa
G
MCP-1
IL-6
MCP-1 protein level (ng/ml)
IL-6 protein level (ng/ml)
H
I
Macrophage migration assays
Relative numbers of migration cells

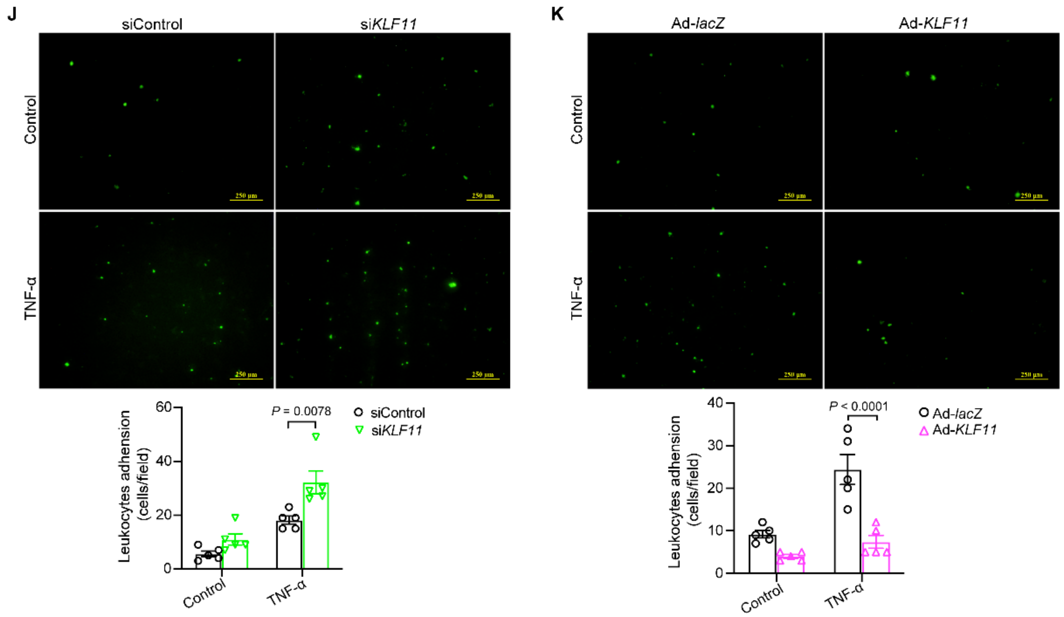

Supplementary Fig. 7: KLF11 suppresses endothelial cell inflammatory activation

A-**B**. HAECs were infected with adenovirus-short hairpin RNA mediated *KLF11* knockdown (Ad-sh*KLF1*, n=4) or *lacZ* (Ad-sh*lacZ*, n=3). After 48h, the total RNA was extracted for RNA sequencing. **A**. The positive enrichment in the inflammatory response (upper panel) and TNF-α signaling *via* NF-κB (lower panel) pathways is shown by GSEA plots (Ad-sh*KLF11* vs. Ad-sh*lacZ*). **B**. The differentially expressed genes (Ad-sh*KLF11* vs. Ad-sh*lacZ*) are shown as a volcano plot. x axis, log2FoldChange (Ad-sh*KLF11* vs. Ad-sh*lacZ*). Green dots, log2FoldChange < -0.5, Red dots, log2Fold Change >0.5. Grey dots, -0.5 < log2FoldChange < 0.5. **C**-**H**. HAECs were infected with Ad-sh*lacZ*, Ad-sh*KLF11*, Ad-*GFP* or Ad-*KLF11* (10MOI). After 48h, the cells were stimulated with TNF-α (2 ng/ml) for 4h (**C**-**F**) or 6h (**G**-**H**). **C** and **E**. Quantitative real-time PCR to detect the mRNA levels of *KLF11*, *VCAM1*, *ICAM1*, *SELE*, *CCL2,* and *IL-6*. The experiment was performed 4 times independently. **D** and **F**. Representative western blot and quantification of the expression of KLF11, VCAM1, and E-selectin. The experiment was performed 4 times independently. **G**-**H**. ELISA measurement of the secretion of MCP-1 and IL-6 in the cell culture medium. **I**. Macrophage migration assays. HAECs were infected with Ad-*lacZ* or Ad-*KLF11* (10 MOI). After 48h, the cells were stimulated with TNF-α (2

ng/ml) for 1h, and then cultured in fresh opti-MEM medium for 4h. Macrophage migration was induced by conditioned medium and measured with the Cultrex® 96 well cell migration assay kit. ng/ml) for 4h. **J-K**. HAECs were transfected with non-target siRNA (siControl), *KLF11* siRNA (si*KLF11*) or infected with Ad-*lacZ*, Ad-*KLF11* (10 MOI). After 48h, the cells were stimulated with TNF-α (2 ng/ml) for 4h. Activated ECs were incubated with GFP-expressing THP-1 cells for 30 min and the adhered cells were evaluated in 9 high power random fields per well. Data are presented as mean±SEM. Two-way ANOVA followed by Holm-Sidak post hoc analysis for C-K.

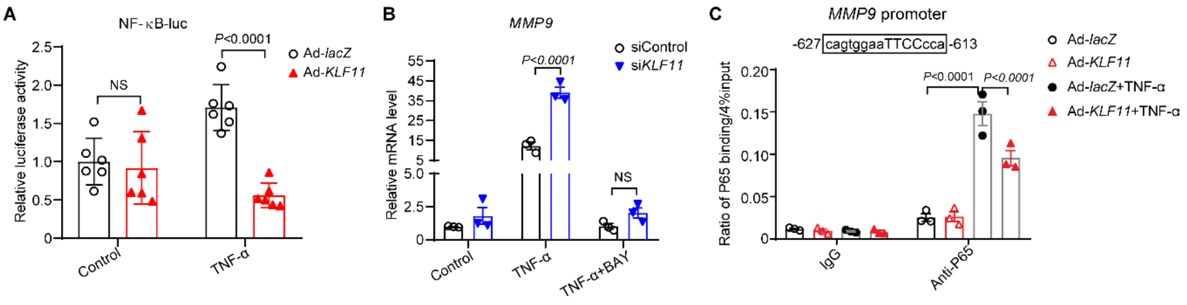

Supplementary Fig. 8: KLF11 reduces *MMP9* expression by inhibiting NF-κB activity in human endothelial cells. A. KLF11 inhibits NF-κB activity by luciferase assay. Human aortic endothelial cells (HAECs) were transfected with NF-κB-luc (the luciferase reporter construct) for 8 hours and then infected with Ad-*lacZ* or Ad-*KLF11* (10 MOI). After 24h, the cells were treated with TNF-α (2 ng/ml) for 12 hours, and the NF-κB activity was subsequently detected by dual-luciferase assay and normalized against *Renilla* activity. **B**. HAECs were transfected with siControl or si*KLF11*. After 48 hours, the cells were pretreated with NF-κB signaling pathway inhibitor BAY11-7082 (BAY, 5 μm) for 1 hour before stimulated with TNF-α (2 ng/ml) for 4 hours. *MMP9* mRNA abundance was determined by qPCR. **C**. HAECs were infected with Ad-*lacZ* or Ad-*KLF11* (10 MOI). After 48 hours, the cells were treated with TNF-α (2 ng/ml) for 1 hour. CHIP assays were performed using an antibody against P65 or normal rabbit IgG, which serves as the control. The binding of P65 to the promoter of *MMP9* was determined by qPCR. Data are presented as mean±SEM. Two-way ANOVA followed by Holm-Sidak post hoc analysis for A-C.

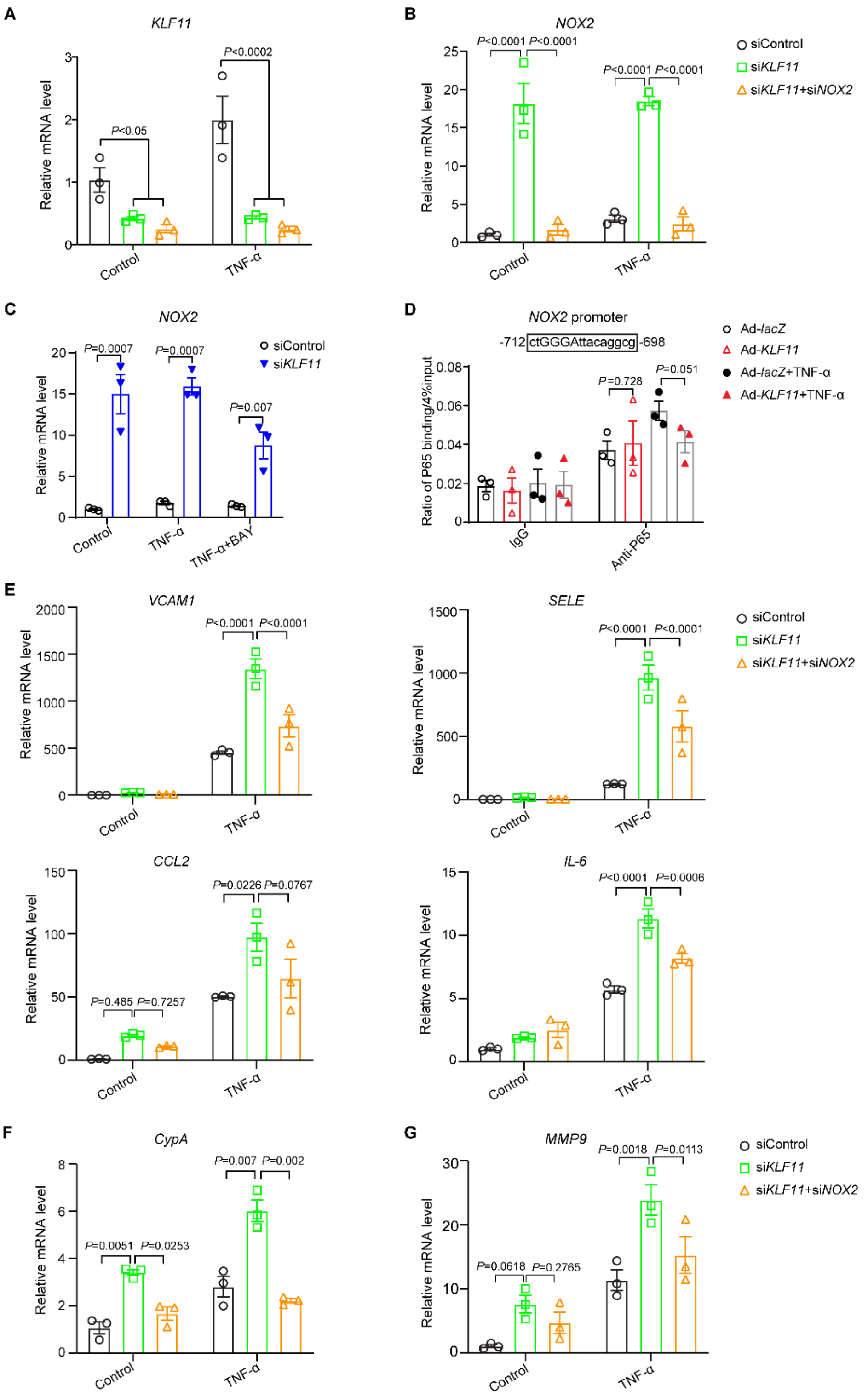

Supplementary Fig. 9: NOX2 inhibition partially suppresses the upregulation of inflammatory genes in *KLF11* knockdown HAECs. A-B and E-G. HAECs were transfected with siControl, si*KLF11,* or si*KLF11*+si*NOX2* (*NOX2* siRNA). After 48h, they were stimulated

with TNF-α (2ng/ml) for 24h. Quantitative real-time PCR to examine the mRNA levels of *KLF11*, *NOX2*, *VCAM1*, *SELE*, *CCL2*, *IL-6*, *CypA,* and *MMP9*. **C**. HAECs were transfected with siControl or si*KLF11*. After 48 hours, the cells were pretreated with NF-κB signaling pathway inhibitor BAY11-7082 (BAY, 5 μm) for 1 hour before stimulated with TNF-α (2 ng/ml) for 4 hours. *NOX2* mRNA abundance was determined by qPCR. **D**. HAECs were infected with Ad-*lacZ* or Ad-*KLF11* (10 MOI). After 48 hours, the cells were treated with TNF-α (2 ng/ml) for 1 hour. CHIP assays were performed using an antibody against P65 or normal rabbit IgG, which serves as the control. The binding of P65 to the promoters of *NOX2* was determined by qPCR. Data are presented as mean±SEM. Two-way ANOVA followed by Holm-Sidak post hoc analysis for A-G.

Supplementary Table 1: List of aortic aneurysm patient information

Sample #	Date of Surgery	Sex	Age (y)	BMI	Race	Description	Western blot
1	6/5/2019	M	37	N/A	Caucasion	Heart transplant-donor	yes
2	6/17/2019	M	28	N/A	Caucasion	Heart transplant-donor	yes
3	7/13/2019	M	49	N/A	Caucasion	Heart transplant-donor	yes
4	8/25/2019	M	40	N/A	Caucasion	Heart transplant-donor	yes
5	4/15/2019	M	57	22.91	Caucasion	Aortic root aneurysm	yes
6	4/23/2019	M	66	21.89	Caucasion	Aortic root aneurysm; ascending aortic aneurysm; aortic arch aneurysm	yes
7	5/15/2019	M	43	28.14	Caucasion	Bicuspid aortic valve, Sievers type 1; aortic stenosis; aortic insufficiency; native valve endocarditis; mitral valve fibroelastoma	yes
8	6/5/2019	M	44	35.4	Caucasion	Bicuspid aortic valve, Sievers type 0; aortic dissection; aortic root aneurysm; ascending aortic aneurysm	yes
9	6/17/2019	M	56	29.23	Caucasion	Aortic root aneurysm; ascending aortic aneurysm	yes

SBP: systolic blood pressure		
DBP: diastolic blood pressure		
TG: triglyceride		
TC: total cholesterol		
LDL: low-density lipoprotein		
HDL: high-density lipoprotein		

Supplementary Table 2: GSEA-Hallmark pathways report for RNA-seq data on Ad-sh*KLF11* vs. Ad-sh*lacZ* infected HAECs

Name	Size	ES	NES	NOM *p*-val	FDR *q*-val	FWER *p*-val
Hallmark_Interferon alpha response	95	0.78	1.45	0	0.119	0.072
Hallmark_Coagulation	137	0.55	1.43	0	0.091	0.136
Hallmark_Notch signaling	32	0.61	1.38	0	0.151	0.304
Hallmark_Interferon gamma response	197	0.69	1.38	0	0.121	0.304
Hallmark_Hedgehog signaling	36	0.61	1.36	0.076	0.114	0.304
Hallmark_Apical surface	44	0.57	1.35	0	0.126	0.365
Hallmark_TNFα signaling via NFκB	200	0.52	1.35	0	0.118	0.365
Hallmark_Wnt beta-catenin signaling	42	0.57	1.35	0.136	0.107	0.365
Hallmark_Inflammatory response	199	0.59	1.31	0	0.161	0.415
Hallmark_Allograft rejection	198	0.51	1.28	0	0.202	0.479
Hallmark_Complement	200	0.5	1.28	0	0.199	0.479
Hallmark_Kras signaling up	199	0.52	1.27	0	0.199	0.479
Hallmark_Cholesterol homeostasis	74	0.5	1.27	0.179	0.191	0.479
Hallmark_Kras signaling down	198	0.43	1.22	0.044	0.275	0.66
Hallmark_Bile acid metabolish	112	0.42	1.21	0.044	0.269	0.66
Hallmark_Adipogenesis	198	0.4	1.21	0.178	0.257	0.686
Hallmark_IL6-JAK-STAT3 signaling	87	0.61	1.2	0	0.261	0.686
Hallmark_Apoptosis	161	0.42	1.18	0.141	0.271	0.686
Hallmark_Heme metabolism	198	0.43	1.16	0.203	0.291	0.686
Hallmark_PI3K-AKT-mTOR signaling	105	0.41	1.14	0.188	0.303	0.686
Hallmark_IL2-STAT5 signaling	199	0.43	1.12	0.05	0.323	0.713
Hallmark_Angiogenesis	36	0.46	1.03	0.366	0.494	0.735
Hallmark_Protein secretion	96	0.38	1.02	0.538	0.512	0.769
Hallmark_Apical junction	200	0.46	0.99	0.659	0.536	0.846
Hallmark_Epithelial mesenchymal transition	199	0.38	0.99	0.449	0.516	0.846
Hallmark_UV response up	158	0.35	0.99	0.417	0.515	0.876
Hallmark_TGF beta signaling	54	0.42	0.99	0.538	0.501	0.876
Hallmark_Fatty acid metabolism	156	0.32	0.95	0.547	0.566	0.876
Hallmark_Estrogen response early	199	0.33	0.94	0.635	0.582	0.912
Hallmark_Peroxisome	104	0.34	0.92	0.577	0.591	0.912
Hallmark_UV response down	143	0.37	0.91	0.511	0.588	0.912
Hallmark_P53 pathway	199	0.32	0.88	0.79	0.645	0.945
Hallmark_Myogenesis	197	0.32	0.87	0.718	0.652	0.945

Hallmark_Myc targets V1	199	-0.59	-1.38	0.165	0.313	0.174
Hallmark_E2F targets	200	-0.63	-1.37	0.128	0.189	0.253
Hallmark_Reactive oxygen species pathway	47	-0.56	-1.32	0.079	0.244	0.304
Hallmark_Spermatogenesis	133	-0.46	-1.29	0	0.273	0.418
Hallmark_Unfolded protein response	112	-0.45	-1.15	0.202	0.515	0.662
Hallmark_G2M checkpoint	199	-0.54	-1.14	0.156	0.484	0.698
Hallmark_Mtorc1 signaling	200	-0.41	-1.13	0.213	0.459	0.782
Hallmark_Myc targets V2	58	-0.45	-1.11	0.3	0.456	0.812
Hallmark_Xenobiotic metabolism	199	-0.35	-1.04	0.267	0.574	0.865
Hallmark_Oxidative phosphorylation	200	-0.37	-1.04	0.406	0.531	0.865
Hallmark_Androgen response	100	-0.37	-1	0.448	0.603	0.887
Hallmark_Estrogen response late	199	-0.35	-1	0.443	0.558	0.887
Hallmark_DNA repair	150	-0.35	-0.99	0.453	0.534	0.915
Hallmark_Hypoxia	199	-0.36	-0.99	0.541	0.504	0.915
Hallmark_Glycolysis	199	-0.32	-0.9	0.7	0.714	1
Hallmark_Pancreas beta cells	40	-0.34	-0.84	0.691	0.799	1
Hallmark_Mitotic spindle	197	-0.37	-0.83	0.795	0.767	1

ES: enrichment score
NES: normalized enrichment score
NOM *p*-val: nominal *p*-value
FDR *q*-val: *p*-value adjusted for the False Discovery Rate
FWER *p*-val: p-value adjusted for the Familywise Error Rate

Supplementary Table 3: Gene expression profile of differentially expressed inflammatory genes from RNA-seq of Ad-sh*KLF11* vs. Ad-sh*lacZ* in HAECs

ensembl_gene_id	external_gene_name	baseMean	log2FoldChange	lfcSE	stat	pvalue	padj
ENSG00000163739	CXCL1	2565.252666	1.366229961	0.097090996	14.07164434	5.67E-45	2.15E-43
ENSG00000081041	CXCL2	462.9932126	3.105737018	0.132908861	23.36741888	9.17E-121	1.96E-118
ENSG00000163734	CXCL3	136.6761429	2.63020667	0.183023628	14.37085859	7.88E-47	3.15E-45
ENSG00000163735	CXCL5	94.11236054	4.907758766	0.364453876	13.46606274	2.48E-41	8.25E-40
ENSG00000124875	CXCL6	1286.564648	1.570846775	0.126038662	12.46321371	1.18E-35	3.17E-34
ENSG00000169245	CXCL10	13.15541345	7.372918207	1.317150092	5.597629496	2.17E-08	1.15E-07
ENSG00000169248	CXCL11	71.02137311	2.8307726	0.314230529	9.008585534	2.09E-19	2.48E-18
ENSG00000108702	CCL1	2.19499265	3.614825581	1.640909007	2.202940909	0.027598914	0.057478962
ENSG00000108691	CCL2	1538.487383	1.405099693	0.107384652	13.08473475	4.03E-39	1.24E-37
ENSG00000108688	CCL7	3.253676306	4.357208512	1.460520238	2.98332635	0.002851338	0.007427671
ENSG00000115009	CCL20	34.38929382	1.642634182	0.333000011	4.932835211	8.10E-07	3.62E-06
ENSG00000274736	CCL23	73.28303068	1.951232681	0.222125021	8.784389414	1.57E-18	1.78E-17
ENSG00000115008	IL1A	65.21024527	1.972004321	0.269784724	7.309547753	2.68E-13	2.18E-12
ENSG00000125538	IL1B	3.761066833	3.932027322	1.453384017	2.705429038	0.006821622	0.016464663
ENSG00000104432	IL7	65.59341683	1.410370875	0.24126966	5.845620511	5.05E-09	2.84E-08
ENSG00000136634	IL10	0.241149029	-1.045453684	3.816920368	-0.273899789	0.784161634	NA
ENSG00000172349	IL16	32.99260682	4.749843513	0.703063559	6.755923345	1.42E-11	1.00E-10
ENSG00000167604	NFKBID	41.3743643	1.198219244	0.377570622	3.173497017	0.001506144	0.004124153
ENSG00000232810	TNF	2.726591696	3.146982225	1.50234469	2.094713847	0.036196438	0.072973877
ENSG00000186827	TNFRSF4	46.75944798	2.203081885	0.326083378	6.756191919	1.42E-11	1.00E-10
ENSG00000049249	TNFRSF9	32.51756188	2.393138846	0.405586519	5.900439816	3.63E-09	2.07E-08
ENSG00000161955	TNFSF13	111.2940127	1.2871249	0.318411575	4.042330751	5.29E-05	0.000186675
ENSG00000181634	TNFSF15	80.40617247	2.31521703	0.325042176	7.122820362	1.06E-12	8.17E-12
ENSG00000162692	VCAM1	393.0942694	0.908428558	0.12398955	7.326654218	2.36E-13	1.92E-12
ENSG00000090339	ICAM1	3401.72318	1.159224457	0.082740396	14.01038077	1.35E-44	5.04E-43
ENSG00000007908	SELE	0.643048491	3.070134616	3.332927738	0.921152469	0.35697083	0.001465252

Supplementary Table 4: GSEA-GO pathways report for RNA-seq data on Ad-sh*KLF11* vs. Ad-sh*lacZ* infected HAECs

Name	Size	ES	NES	NOM *p*-val	FDR *q*-val	FWER *p*-val
GO_ANTIMICROBIAL_HUMORAL_IMMUNE_RESPONSE_MEDIATED_BY_AN	71	0.7312	1.7550	0	0.0694	0.069
GO_REGULATION_OF_HUMORAL_IMMUNE_RESPONSE	125	0.6787	1.7439	0	0.0508	0.1
GO_RESPONSE_TO_TYPE_I_INTERFERON	93	0.7013	1.7395	0	0.0390	0.114
GO_NEUTROPHIL_MIGRATION	111	0.6910	1.7315	0	0.0395	0.152
GO_STRESS_RESPONSE_TO_METAL_ION	16	0.9295	1.7299	0	0.0332	0.158
GO_RESPONSE_TO_CHEMOKINE	93	0.6915	1.7136	0	0.0416	0.228
GO_DEFENSE_RESPONSE_TO_VIRUS	238	0.6288	1.6979	0	0.0583	0.353
GO_LUNG_MORPHOGENESIS	50	0.7445	1.6748	0	0.0884	0.512
GO_RESPONSE_TO_INTERFERON_GAMMA	191	0.6346	1.6705	0	0.0874	0.555
GO_POSITIVE_REGULATION_OF_MYELOID_LEUKOCYTE_DIFFERENTIATI(	53	0.7167	1.6647	0.0014	0.0929	0.612
GO_ANTIMICROBIAL_HUMORAL_RESPONSE	116	0.6604	1.6557	0	0.1072	0.7
GO_GRANULOCYTE_MIGRATION	133	0.6417	1.6460	0	0.1221	0.778
GO_HUMORAL_IMMUNE_RESPONSE	334	0.5942	1.6275	0	0.1736	0.901
GO_ANTIGEN_PROCESSING_AND_PRESENTATION_OF_ENDOGENOUS_A	20	0.8352	1.6257	0	0.1674	0.905
GO_POSITIVE_REGULATION_OF_OSTEOCLAST_DIFFERENTIATION	24	0.8075	1.6206	0	0.1744	0.935
GO_MEMBRANE_PROTEIN_PROTEOLYSIS	58	0.6759	1.5914	0	0.2921	0.993
GO_ZINC_ION_HOMEOSTASIS	37	0.7271	1.5899	0.0070	0.2826	0.995
GO_MEMBRANE_PROTEIN_ECTODOMAIN_PROTEOLYSIS	41	0.7129	1.5897	0.0029	0.2678	0.995
GO_COLLAGEN_CATABOLIC_PROCESS	46	0.6985	1.5868	0.0028	0.2675	0.997
GO_RESPONSE_TO_INTERFERON_ALPHA	20	0.8185	1.5854	0.0044	0.2610	0.997
GO_LYMPHOCYTE_MIGRATION	105	0.6305	1.5841	0	0.2549	0.997
GO_INTERFERON_GAMMA_MEDIATED_SIGNALING_PATHWAY	86	0.6457	1.5778	0	0.2736	0.998
GO_EOSINOPHIL_MIGRATION	24	0.7841	1.5654	0.0046	0.3320	1
GO_REGULATION_OF_VIRAL_ENTRY_INTO_HOST_CELL	30	0.7400	1.5571	0.0029	0.3700	1
GO_NEGATIVE_REGULATION_OF_PEPTIDE_SECRETION	131	0.6033	1.5552	0	0.3686	1
GO_INTERFERON_GAMMA_SECRETION	18	0.8129	1.5534	0.0046	0.3652	1
GO_REGULATION_OF_DEFENSE_RESPONSE_TO_VIRUS	71	0.6446	1.5524	0.0026	0.3583	1
GO_EOSINOPHIL_CHEMOTAXIS	20	0.8012	1.5520	0.0119	0.3480	1
GO_RESPONSE_TO_VIRUS	321	0.5656	1.5518	0	0.3369	1
GO_LEUKOCYTE_MEDIATED_CYTOTOXICITY	105	0.6143	1.5491	0	0.3418	1
GO_LYMPHOCYTE_CHEMOTAXIS	61	0.6506	1.5410	0.0066	0.3810	1
GO_NEGATIVE_REGULATION_OF_CYTOKINE_SECRETION	63	0.6483	1.5367	0	0.3973	1
GO_T_CELL_MIGRATION	61	0.6540	1.5360	0.0054	0.3894	1
GO_MYD88_INDEPENDENT_TOLL_LIKE_RECEPTOR_SIGNALING_PATHWA	32	0.7154	1.5333	0.0101	0.3935	1

GO_NEGATIVE_REGULATION_OF_VIRAL_PROCESS	95	0.6168	1.5312	0	0.3960	1
GO_CELLULAR_RESPONSE_TO_ZINC_ION	22	0.7794	1.5288	0.0057	0.3998	1
GO_REGULATION_OF_LIPID_CATABOLIC_PROCESS	52	0.6601	1.5247	0.0093	0.4153	1
GO_EPITHELIAL_TUBE_BRANCHING_INVOLVED_IN_LUNG_MORPHOGENE	29	0.7303	1.5214	0.0090	0.4253	1
GO_NEGATIVE_REGULATION_OF_MULTI_ORGANISM_PROCESS	173	0.5794	1.5208	0.0012	0.4185	1
GO_POSITIVE_REGULATION_OF_INTERLEUKIN_2_PRODUCTION	30	0.7124	1.5138	0.0156	0.4595	1
GO_INTERFERON_BETA_PRODUCTION	49	0.6644	1.5125	0.0081	0.4572	1
GO_ANTIBACTERIAL_HUMORAL_RESPONSE	43	0.6740	1.5060	0.0125	0.4938	1
GO_NEGATIVE_REGULATION_OF_INTERLEUKIN_10_PRODUCTION	18	0.7897	1.4987	0.0123	0.5367	1
GO_NEGATIVE_REGULATION_OF_DEFENSE_RESPONSE	193	0.5642	1.4951	0	0.5525	1
GO_I_KAPPAB_PHOSPHORYLATION	18	0.7894	1.4941	0.0199	0.5480	1
GO_CAMERA_TYPE_EYE_PHOTORECEPTOR_CELL_DIFFERENTIATION	19	0.7662	1.4921	0.0219	0.5520	1
GO_NEGATIVE_REGULATION_OF_INFLAMMATORY_RESPONSE	131	0.5790	1.4889	0	0.5671	1
GO_REGULATION_OF_OLIGODENDROCYTE_DIFFERENTIATION	32	0.6935	1.4853	0.0289	0.5850	1
GO_I_KAPPAB_KINASE_NF_KAPPAB_SIGNALING	259	0.5537	1.4851	0	0.5746	1
GO_REGULATION_OF_IMMUNE_EFFECTOR_PROCESS	445	0.5349	1.4845	0	0.5682	1
GO_RETINA_VASCULATURE_DEVELOPMENT_IN_CAMERA_TYPE_EYE	18	0.7849	1.4843	0.0186	0.5584	1
GO_AORTIC_VALVE_DEVELOPMENT	28	0.7074	1.4843	0.0240	0.5481	1
GO_MULTI_ORGANISM_CELLULAR_PROCESS	48	0.6529	1.4809	0.0110	0.5647	1
GO_REGULATION_OF_RESPONSE_TO_BIOTIC_STIMULUS	135	0.5801	1.4803	0	0.5586	1
GO_REGULATION_OF_DEFENSE_RESPONSE_TO_VIRUS_BY_HOST	37	0.6741	1.4795	0.0113	0.5551	1
GO_MONOCYTE_CHEMOTAXIS	62	0.6284	1.4791	0.0082	0.5478	1
GO_REGULATION_OF_CD4_POSITIVE_ALPHA_BETA_T_CELL_ACTIVATION	57	0.6316	1.4755	0.0055	0.5662	1
GO_RESPONSE_TO_ZINC_ION	54	0.6369	1.4752	0.0070	0.5588	1
GO_NEGATIVE_REGULATION_OF_VIRAL_TRANSCRIPTION	24	0.7277	1.4717	0.0176	0.5762	1
GO_NEGATIVE_REGULATION_OF_TYPE_I_INTERFERON_PRODUCTION	44	0.6574	1.4711	0.0198	0.5717	1
GO_TUMOR_NECROSIS_FACTOR_SUPERFAMILY_CYTOKINE_PRODUCTIO	139	0.5691	1.4708	0.0036	0.5647	1
GO_REGULATION_OF_CELL_KILLING	89	0.5958	1.4698	0.0112	0.5627	1
GO_POSITIVE_REGULATION_OF_TUMOR_NECROSIS_FACTOR_SUPERFA	78	0.5970	1.4693	0.0117	0.5582	1
GO_REGULATION_OF_ODONTOGENESIS	26	0.7216	1.4675	0.0217	0.5625	1
GO_LEUKOCYTE_CHEMOTAXIS	214	0.5548	1.4643	0	0.5771	1
GO_DETECTION_OF_LIGHT_STIMULUS	75	0.6061	1.4640	0.0040	0.5704	1
GO_POSITIVE_REGULATION_OF_RESPONSE_TO_EXTERNAL_STIMULUS	296	0.5367	1.4635	0	0.5656	1
GO_CD4_POSITIVE_ALPHA_BETA_T_CELL_ACTIVATION	86	0.5969	1.4626	0.0038	0.5634	1
GO_LYTIC_VACUOLE_ORGANIZATION	60	0.6236	1.4617	0.0123	0.5627	1
GO_REGULATION_OF_NEUTROPHIL_MIGRATION	32	0.6939	1.4615	0.0131	0.5566	1

GO_PHOTOTRANSDUCTION	61	0.6192	1.4596	0.0117	0.5619	1
GO_CELL_KILLING	155	0.5591	1.4574	0.0024	0.5701	1
GO_REGULATION_OF_LIPOPROTEIN_PARTICLE_CLEARANCE	20	0.7387	1.4566	0.0407	0.5681	1
GO_T_CELL_MEDIATED_CYTOTOXICITY	48	0.6427	1.4565	0.0153	0.5617	1
GO_INTERLEUKIN_6_BIOSYNTHETIC_PROCESS	22	0.7374	1.4563	0.0274	0.5555	1
GO_REGULATION_OF_NEUROINFLAMMATORY_RESPONSE	31	0.6867	1.4543	0.0292	0.5627	1
GO_REGULATION_OF_CD4_POSITIVE_ALPHA_BETA_T_CELL_DIFFERENT	46	0.6455	1.4536	0.0234	0.5602	1
GO_MAINTENANCE_OF_GASTROINTESTINAL_EPITHELIUM	20	0.7516	1.4536	0.0291	0.5532	1
GO_NEGATIVE_REGULATION_OF_GLIAL_CELL_DIFFERENTIATION	23	0.7316	1.4535	0.0333	0.5473	1
GO_BLOOD_COAGULATION_INTRINSIC_PATHWAY	18	0.7680	1.4530	0.0242	0.5439	1
GO_MYELOID_LEUKOCYTE_MIGRATION	196	0.5514	1.4516	0.0011	0.5471	1
GO_DETECTION_OF_EXTERNAL_BIOTIC_STIMULUS	18	0.7502	1.4508	0.0313	0.5457	1
GO_INTERLEUKIN_2_PRODUCTION	63	0.6117	1.4488	0.0189	0.5529	1
GO_REGULATION_OF_T_CELL_APOPTOTIC_PROCESS	35	0.6802	1.4482	0.0265	0.5507	1
GO_REGULATION_OF_SYSTEMIC_ARTERIAL_BLOOD_PRESSURE_BY_CIF	19	0.7489	1.4430	0.0304	0.5810	1
GO_OUTER_DYNEIN_ARM_ASSEMBLY	17	0.7647	1.4426	0.0230	0.5764	1
GO_POSITIVE_REGULATION_OF_CALCIUM_ION_TRANSMEMBRANE_TRAI	37	0.6560	1.4423	0.0260	0.5717	1
GO_NOREPINEPHRINE_SECRETION	17	0.7649	1.4416	0.0369	0.5702	1
GO_TISSUE_REMODELING	155	0.5468	1.4397	0.0047	0.5771	1
GO_CYTOPLASMIC_PATTERN_RECOGNITION_RECEPTOR_SIGNALING_P/	27	0.7020	1.4388	0.0147	0.5766	1
GO_CELL_CHEMOTAXIS	287	0.5282	1.4386	0	0.5721	1
GO_REGULATION_OF_DEFENSE_RESPONSE_TO_BACTERIUM	18	0.7566	1.4377	0.0471	0.5719	1
GO_RESPIRATORY_SYSTEM_PROCESS	28	0.6950	1.4367	0.0302	0.5724	1
GO_NEGATIVE_REGULATION_OF_VIRAL_GENOME_REPLICATION	57	0.6178	1.4343	0.0148	0.5836	1
GO_FATTY_ACID_DERIVATIVE_BIOSYNTHETIC_PROCESS	60	0.6094	1.4340	0.0214	0.5796	1
GO_T_CELL_APOPTOTIC_PROCESS	48	0.6390	1.4331	0.0125	0.5802	1
GO_CELL_DIFFERENTIATION_IN_SPINAL_CORD	52	0.6284	1.4320	0.0148	0.5826	1
GO_CELLULAR_EXTRAVASATION	55	0.6200	1.4316	0.0150	0.5792	1

ES: enrichment score						
NES: normalized enrichment score						
NOM p-val: nominal p-value						
FDR q-val: p-value adjusted for the False Discovery Rate						
FWER p-val: p-value adjusted for the Familywise Error Rate						

Supplementary Table 5: Gene expression profile of differentially expressed proteinase genes from RNA-seq of Ad-sh*KLF11* vs. Ad-sh*lacZ* in HAECs

ensembl_gene_id	gene_name	baseMean	log2FoldChange	lfcSE	stat	pvalue	padj
ENSG00000087245	MMP2	20106.4988	0.292513997	0.07812978	3.743950004	0.00018115	0.00058558
ENSG00000149968	MMP3	0.19548441	-0.303493791	3.81692037	-0.079512738	0.9366248	NA
ENSG00000137673	MMP7	3.99560583	1.714170122	0.96890726	1.769178737	0.07686405	0.14029021
ENSG00000100985	MMP9	0.98383784	1.63693399	2.0800973	0.786950684	4.31E-01	4.99E-02
ENSG00000137745	MMP13	0.08529716	0.637171384	3.81692037	0.166933371	0.86742248	NA
ENSG00000157227	MMP14	225.609908	-0.454736767	0.31055899	-1.46425248	1.43E-01	0.23661231
ENSG00000102996	MMP15	423.702195	2.190636102	0.10844918	20.19965497	9.86E-91	1.28E-88
ENSG00000154734	ADAMTS1	3306.58991	-0.873660184	0.08603602	-10.15458598	3.16E-24	4.95E-23
ENSG00000156140	ADAMTS3	114.594837	0.798207702	0.19164914	4.164942894	3.11E-05	1.14E-04
ENSG00000158859	ADAMTS4	1995.00497	1.180173909	0.14716614	8.019330396	1.06E-15	1.01E-14
ENSG00000136378	ADAMTS7	2782.64115	0.917347097	0.07528413	12.18513252	3.73E-34	9.4E-33
ENSG00000163638	ADAMTS9	1947.77841	1.043311964	0.10649897	9.796451546	1.17E-22	1.68E-21

Supplementary Table 6. Sequences of the primers used for real-time PCR

Name	Forward	Reverse
human *KLF11*	GACACACACCTCACGGACAG	ATCATCTGGCAAAGGACAGG
human *VCAM1*	CGAACCCAAACAAAGGCAGA	ACAGGATTTTCGGAGCAGGA
human *ICAM1*	AAGATCAAATGGGGCTGGGA	AATGTATGTGGGTGGGGAGG
human *SELE*	ACTTTCTGCTGCTGGACTCT	TAGTTCCCCAGATGCACCTG
human *CCL2*	CCGAGAGGCTGAGACTAA	GAAGGTGGCTGCTATGAG
human *IL-6*	GTACATCCTCGACGGCATC	TTTCACCAGGCAAGTCTCC
human *ADAMTS1*	AAAGCTTCCTTTGGGAGTGG	GTGGAATCTGGGCTACATGG
human *MMP2*	TATTTGATGGCATCGCTCAG	GCCTCGTATACCGCATCAAT
human *MMP3*	AACCTGTCCCTCCAGAACCT	GGAAGAGATGGCCAAAATGA
human *MMP9*	TGGGAAGTACTGGAGATTCT	CCTGTGTACACCCACACCTG
human *MMP13*	AACATCCAAAAACGCCAGAC	GGAAGTTCTGGCCAAAATGA
human *MMP14*	GAGCTCAGGGCAGTGGATAG	GGTAGCCCGGTTCTACCTTC
human *NOX1*	CGTCTGCTCTCTGCTTGAAT	TTGTGGAAGGTGAGGTTGTG
human *NOX2*	AACTGGGCTGTGAATGAGGG	CCAGTGCTGACCCAAGAAGT
human *NOX4*	GCAGGAGAACCAGGAGATTG	ACGTCAGCAGCATGTAGAAG
human *NOX5*	GCAGGTACAGAGTGGGGTG	GGTCAGGTTCTCCATGACTCC
human *ACTA2*	CTTGAGAAGAGTTACGAGTTG	GATGCTGTTGTAGGTGGTT
human *CNN1*	AGGCTCCGTGAAGAAGAT	CCTGTGTATGGTTGGTGTT
Human *GAPDH*	CCAAGGAGTAAGACCCCTGG	TGGTTGAGCACAGGGTACTT
